# Potentially Toxic Substances and Associated Risks in Soils Affected by Wildfires: A Review

**DOI:** 10.3390/toxics10010031

**Published:** 2022-01-11

**Authors:** Maria Luisa Fernandez-Marcos

**Affiliations:** 1 Department of Soil Science and Agricultural Chemistry, Universidad de Santiago de Compostela, 27002 Lugo, Spain; mluisa.fernandez@usc.es; Tel.: +34-982823119; 2 Institute of Agricultural Biodiversity and Rural Development, Universidad de Santiago de Compostela, 27002 Lugo, Spain

**Keywords:** fire, soil quality, soil pollution, heavy metals, trace elements, PAH, pyrogenic compounds, pyrogenic organic matter, charcoal, ash, soil ecotoxicity

## Abstract

The presence of toxic substances is one of the major causes of degradation of soil quality. Wildfires, besides affecting various chemical, physical, and biological soil properties, produce a mixture of potentially toxic substances which can reach the soil and water bodies and cause harm to these media. This review intends to summarise the current knowledge on the generation by wildfires of potentially toxic substances, their effects on soil organisms, and other associated risks, addressing the effects of fire on metal mobilisation, the pyrolytic production of potentially toxic compounds, and the detoxifying effect of charcoal. Numerous studies ascertained inhibitory effects of ash on seed germination and seedling growth as well as its toxicity to soil and aquatic organisms. Abundant publications addressed the mobilisation of heavy metals and trace elements by fire, including analyses of total concentrations, speciation, availability, and risk of exportation to water bodies. Many publications studied the presence of polycyclic aromatic hydrocarbons (PAH) and other organic pollutants in soils after fire, their composition, decline over time, the risk of contamination of surface and ground waters, and their toxicity to plants, soil, and water organisms. Finally, the review addresses the possible detoxifying role of charcoal in soils affected by fire.

## 1. Introduction

Soil is a crucial component of ecosystems and plays a major role in ecosystem services. In addition to their function in plant growth and biomass production, soils are involved in water, carbon, and nutrient cycling, regulate the climate, preserve biodiversity, process wastes, and help maintain the water and air quality. The provision of ecosystem services by the soil decreases with decreasing soil quality. Therefore, soil degradation is a major challenge for ecosystem sustainability [1]. Among the major factors producing deterioration of soil quality, it is necessary to highlight the presence of toxic substances, which have a deleterious effect on soil biota, enzymatic activity and on the protective role of soil on the quality of water, air, and vegetation.

Wildfires are among the main sources of disturbance in terrestrial ecosystems, which depends on fire intensity, severity, and recurrence, as well as on the environmental and climatic characteristics of the region [2]. Forest fires can be produced by natural or anthropogenic causes and are estimated to burn 82 million ha per year worldwide [3], while the global burnt land is estimated to be between 420 and 470 million ha per year [4,5]. They can affect various environmental compartments, including soil. Soils can be affected by fire either directly, by the high soil temperatures generated by the fire (which can range between 50 and 1500 °C [6,7]) and combustion processes, or indirectly, through ash deposition, changes in vegetation cover, or post-fire erosion [7]. Major wildfire effects on soil include loss of soil organic matter, deterioration of soil structure, decreased porosity, reduced water and cations retention, increase of soil pH and electrical conductivity, alteration of soil microbial and invertebrates’ communities, increased soil water repellence, decreased infiltration, and increased runoff and erosion [8,9,10,11]. These effects depend on the fire intensity, soil properties, and type of vegetation. The combustion of vegetation and soil organic matter releases heat and a mixture of gaseous and particulate by-products, including toxic substances. The incidence of wildfires is projected to increase in a climate change scenario.

The present review is intended to summarise the current knowledge on the generation by wildfires of potentially toxic substances, their effects on soil organisms, and other associated risks, addressing the effects of fire on metal mobilisation and the pyrolytic production of potentially toxic compounds, as well as the detoxifying effect of charcoal.

## 2. Effects of Ash and Burning on Plants, Soil, and Water Organisms

Ash is the most common residue left by fire on the surface of burnt soils and is composed of mineral substances as well as charred organic matter, including charcoal. It has an alkaline pH, due to the presence of metal oxides and carbonates, and is rich in water-soluble species, including plant nutrients. The reported thicknesses of the ash layer range from near 0 to 50 mm [12]. Depending on the fire temperature, type of burnt vegetation, thickness of the ash layer, or soil texture, ash can either constitute a hydrophobic layer, reducing infiltration, or act as a mulch layer exerting a protective function against soil erosion [12,13,14]. In either case, ash may contain substances capable of adversely affecting soil and water organisms.

### 2.1. Effects of Ash or Burning on Plant Species

Thomas and Wein [15,16] investigated the establishment of jack pine (*Pinus banksiana* Lamb.) on the ash from wildfires in field and laboratory experiments in Canada (Table 1). The authors reported that seedlings did not survive on unleached wood ash but leaching improved germination and survival. They interpreted the results as caused by the high pH of the ash.

Ne’eman et al. [17,18] reported that high levels of ash reduced germination and/or growth of shoot and root in most species studied (*Pinus halepensis*, *Cistus salviifolius*, *C. creticus*, wheat, and radish). According to these authors, the inhibition of germination and growth may be due to a high osmotic potential of the soil solution or to the toxic effect of certain unidentified ions. Moreover, they reported that the harmful effects of ash were higher for *Cistus salviifolius* and *C. creticus* than for *Pinus halepensis*. Therefore, germination in pot experiments resulted in significant reduction of seedlings of both *C. salviifolius* and *P. halepensis* germinated with a thick cover of ash compared to control (up to 94% reduction in *P. halepensis* and 99% in *C. salviifolius*). Shoot growth of *P. halepensis* was not significantly affected by the ash cover, while in *C. salviifolius*, the shoot growth was significantly reduced (up to 93%). Furthermore, low levels of ash had a positive (not significant) effect on pine growth in petri dishes. This shows that *Pinus* is better adapted than *Cistus* to germination and growth in post-fire conditions. In a study carried out in Israel on the recovery of a *Pinus halepensis* Mill. forest after a wildfire, Ne’eman [19] found that pine ash inhibited post-fire seed germination and attributed this effect to the high pH of the ash. According to Henig-Sever [20], extreme pH values are the main factor in inhibiting seed germination by ash, while osmotic potential is of secondary importance. Ne’eman et al. [21] studied the germination of the post-fire pioneer species *Rhus coriaria* L. (Sumack) in *Pinus halepensis* forests in Israel. The germination of this species is restricted to microsites covered by ash under burnt pine trees, where the germination of other species is inhibited. The authors reported that germination increased with an ash cover of 1 cm (1.2 kg m^−2^) and 2 cm (2.4 kg m^−2^) but was inhibited by an ash cover of 5 cm (6 kg m^−2^). The post-fire germination of *R. coriaria* is determined by the balance between the stimulating effects of heat and the ethylene released by wet ash, and the inhibitory effects of the high pH and the high osmotic pressure in the soil under the ash layer. Similarly, Izhaki et al. [22] reported an effect of ash as germination inhibitor for all taxa in the soil seed bank in *Pinus halepensis* forests in Israel and attributed this effect to the high pH.

González-Rabanal and Casal [23] reported lower germination rates of various shrubland seeds, particularly two Ericaceae (*Calluna vulgaris* and *Erica umbellata*), in the presence of ash. They tentatively attributed this effect to the alkaline pH. Reyes and Casal [24] found that the addition of ash solutions did not significantly influence the germination rate of *Pinus pinaster*, *Pinus radiata*, or *Eucalyptus globulus* seeds, whereas the direct application of ash markedly reduced the germinative capacity of *P. pinaster* and *P. radiata* and had a completely inhibitory effect on the *E. globulus* seeds. Several ash effects (high osmotic pressure, high pH, or toxic effects of certain ions) can be the cause of the reduction of germination. Reyes and Casal [25] reported that, in *Pinus sylvestris* L., *Pinus nigra* Arn., *Pinus radiata* D. Don, and *Pinus pinaster* Aiton, the ash produced by forest fires has an inhibiting effect on germination and germination rate decreased as the amount of ash applied increased, especially for *P. sylvestris* and *P. nigra*. However, it appears that ash has little effect on the development of seedlings in the first months of life. By contrast, for *Quercus robur*, *Q. pyrenaica*, and *Q. ilex*, the same authors [26] reported that ash, either applied directly or diluted in water, did not modify the germination rates. Consistent with [24], Escudero et al. [27] found that the application of ash solutions did not have any significant effect on the germination of seeds of *Pinus nigra ssp salzmannii* and *P. sylvestris*. Recently, Reyes et al. [28] analysed the effects of three ash and one black carbon concentration on the germination of the tree species *Acacia dealbata* Link, *A. longifolia* (Andrews) Willd., *A. mearnsii* De Wild., *A. melanoxylon* R. Br., *Pinus nigra* Arnold, *P. pinaster* Aiton, *P. radiata* D. Don, *P. sylvestris* L., *Quercus ilex* L., *Q. pyrenaica* Willd., *Q. robur* L., and *Q. rubra* L. They reported that in six species (including the four species of pine), ash inhibited germination and this effect increased with increasing ash concentration, whereas in another five species (including *Quercus ilex*, *Q. pyrenaica*, and *Q. robur*) germination was not affected by ash or by black carbon. The ash and black carbon from *Ulex europaeus* L. (gorse) stimulated the germination of *Q. rubra*. This stimulation by gorse ash could be related to its richness in N.

Paradelo et al. [29] investigated the phytotoxicity of a forest soil burnt in the laboratory at various temperatures (100–500 °C). The soil was an acid forest soil, rich in organic matter, developed from granite in Galicia, NW Spain. Plant establishment failed when seeds were placed directly on the surface of the burnt soil, whereas the application of a mulch allowed plants to germinate and establish in the burnt soil. The authors discarded pH and salinity as plausible causes of the low plant cover in the burnt soil, since they did not reach very high values. The presence in burnt soil of polycyclic aromatic hydrocarbons does not seem to be the cause of phytotoxicity either, since their concentrations did not reach values high enough to explain a detrimental effect on seed germination and plant growth. Soil heating above 100 °C increased its phytotoxicity, determined by means of a germination-elongation test with *Lepidium sativum* L., pointing to the presence of non-hydrosoluble phytotoxic organic substances, which reached their highest concentrations at around 200 °C. The authors hypothesise that the inhibition of plant growth could result from a combination of the negative conditions created by the soil hydrophobicity and the presence of undetermined phytotoxic substances.

Barroso and Vaverková [30] evaluated the phytotoxicity of a soil affected by a wildfire in the Czech Republic by using phytotoxicity tests. The tests are based on measuring the inhibition of seed germination or root growth of test plants. Surface (0–20 cm) soils were sampled 6 months after the fire. The burnt soil inhibited the root growth of *Lepidium sativum* L. and *Sinapis alba* L. However, the study does not identify the substances responsible for the phytotoxicity.

Overall, most of the reviewed literature has linked the phytotoxic effects of ash to high pH, high osmotic pressure of the soil solution, or the presence of unidentified toxic substances (Table 1).

### 2.2. Effects of Ash and Burning on Soil Microorganisms

Widden and Parkinson [31] investigated the effects of a forest fire on soil microfungi in a *Pinus contorta* forest in Canada. Some fungus species were killed by the heat of the fire. Moreover, the growth of various fungus species was inhibited by aqueous extracts of burnt litter, indicating the presence of a water-soluble substance toxic to fungi produced during the forest fire (Table 2). Díaz-Raviña et al. [32] studied the effects of soil heating on bacterial activity in a forest soil in a laboratory experiment. They reported that heating at 200 °C for one hour decreased bacterial activity. This temperature can be attained in surface soil in low severity fires [11] and is below the ignition temperature of soil organic matter [33]. Díaz-Raviña et al. [32] suggest that the presence of an undetermined toxic substance could be the cause of the prolonged reduced bacterial activity in the heated soils, which is in accordance with findings by Paradelo et al. [29]. Fritze et al. [34] applied extracts from burnt humus to samples of unburnt humus of the same *Myrtillus* type forest and found that the application of these extracts decreased the respiration rate, modified the structure of the microbial community, and caused toxicity to the indicator luminescent bacteria *Vibrio fischeri*. By chemical fractionation of the dissolved organic carbon (DOC), the authors ascertained that the effects of burnt humus extracts were caused by inhibitory compounds of low molecular mass present in the hydrophilic base fraction (which contains proteins, free amino acids and peptides, aromatic amines, and aminosugar polymers). The molecular composition of these compounds was not determined.

Badía and Martí [35] studied the effects of heating and ash addition on the microbiological properties of two soils of semiarid Spain in a laboratory experiment. Topsoil samples (0–15 cm) were heated to 150 °C, 250 °C, and 500 °C. Samples of the soil heated to 250 °C were mixed with black ash (1%, *w*/*w*) from *Cistus clusii* burning. Soil samples were incubated for nine months and soil respiration, biomass-C, bacteria, and fungi numbers were analysed. Heating significantly reduced the basal respiration and biomass C of the gypsiferous soil up to 150 °C, and drastically reduced these at 500 °C. However, in the calcareous soil, the basal respiration and biomass C were stimulated at 150 °C and 250 °C, while reduced at 500 °C. The number of bacteria decreased significantly at 500 °C in both soils, but at intermediate temperatures the behaviour was different in both soils, increasing at 250 °C in the calcareous soil. The number of fungi decreased progressively as the temperature increased in both soils. The addition of black ash increased basal respiration in both soils but did not affect other biological properties. The results showed that deleterious effects on soil biological properties were produced by heating but not by ash addition. The increase of basal respiration was attributed to the addition of organic carbon by black ash. Similar results were reported by Fritze et al. [36], who studied the effect of burning and wood-ash fertilisation on microbiological properties of the soil organic horizon in a *Pinus sylvestris* L. stand in Finland. They found that the fire treatment significantly reduced the total microbial biomass and the fungal biomass by nearly 50%. In contrast, the ash application did not produce any significant change in microbial biomass or fungal biomass. Similarly, the fire treatment caused a decrease of the respiration rate, whereas the addition of ash increased the respiration rate compared to the control plots. The authors concluded that the decreases in soil microbial biomass and respiration in the fire treatment were due to the fire itself and not to the ash. The ash promoted faster mineralisation of soil organic matter.

Deleterious effects of fire on soil microorganisms were attributed in the literature reviewed to high temperatures or to the presence in ash of unidentified toxic substances (Table 2).

### 2.3. Effects of Ash on Other Soil and Aquatic Organisms

Oliveira et al. [37], in a study carried out after a wildfire in a Brazilian savanna (Cerrado *sensu stricto*), evaluated the ecotoxicological effects of ashes on the aquatic snail *Biomphalaria glabrata* and the soil annelid *Enchytraeus* sp. The ash caused a statistically significant decrease in the number of eggs per snail exposed to it (Table 3). The *Enchytraeus* sp. reproduction was also negatively affected when exposed to the ashes from the burnt area. The authors attributed these effects to pH and possibly to other unidentified compounds present in ash.

Several publications studied the ecotoxicological effects of fire ash to aquatic organisms. Brito et al. [38] studied the ecotoxicity of ashes from wildfires in the Cerrado region in central Brazil. These researchers assessed ashes from three wildfires, using three standard aquatic test species (*Ceriodaphnia dubia*, *Danio rerio*, and *Biomphalaria glabrata*). Moreover, total and water-soluble major and trace elements were determined in ash. The ecotoxicity assays showed a strong impact of ash from the three studied areas (Cerrado sensu stricto, pasture, and transition area) on *C. dubia* at low concentrations. Only the transition area presented toxicity to *D. rerio* and no toxicity to *B. glabrata* was observed. The study was not conclusive on which substances in the ash were responsible for the toxicity.

Harper et al. [39] tested the toxicity of ash from various plant species by studying its effects on the indicator aquatic crustacean *Daphnia magna*. Ash was sampled after wildfires, before any rainfall, in Australia, USA, Canada, Spain, and the UK. The ash samples were analysed for 35 PAHs, Al, B, Cu, Fe, Ni, As, Cd, Hg, Pb, and other chemical parameters. Three of the six types of ash tested (Australia, USA, and Canada) showed notable toxicity to *D. magna*. The toxicity was related to high values of pH and electrical conductivity. The authors suggest that pH may have an indirect effect on toxicity, by influencing the dissolution of toxic ash components. Oxyanions such as arsenate, chromate, or vanadate are more strongly adsorbed at acid pH [40,41,42,43], thus being mobilised at alkaline pH. High values of electrical conductivity are associated to high ionic concentrations in solution. High water-soluble concentrations of Mn, Fe, Zn, Pb, Cu, and As did not produce any significant ecotoxicity. The relatively high concentrations of PAH in the ash appeared to produce no observable toxicity on *D. magna*, maybe because they were not bioavailable. Even though this study focused on aquatic environment, the results could be extrapolated to soil systems.

The literature reviewed attributed ash toxicity to soil and water animal species to high pH or electrical conductivity or to the presence of unidentified toxic substances (Table 3).

## 3. Metal Mobilisation

Soils often retain potentially toxic elements from various sources, either lithologic or anthropogenic. Among anthropogenic sources, old mining works [44], burning of fossil fuels, nearby urban settlements [10,45], waste incineration, or industrial activities [46,47] are worth mentioning. Moreover, certain elements, even at low concentration in soil, can accumulate in leaves of various forest trees, so that they reach high concentrations in litter [48,49,50]. When soil is affected by a wildfire, these elements are released, particularly upon burning of forest floor or soil organic matter [51], but also by transformation of minerals bearing the concerning elements [52,53]. Furthermore, burning vegetation releases toxic elements stored therein. The released elements can be deposited on the soil surface or exported to other environmental compartments (atmosphere, water bodies). Potentially toxic elements can accumulate in the ash. Ash can be dispersed by the wind or carried by runoff into streams or rivers. Depending on the element, the mobility of these elements can be hampered or enhanced by an increase in soil pH, which usually occurs as a result of fire [53,54,55]. Elements present in cationic form in aquatic media (Cu, Fe, Mn, Ni, Zn, Pb, Cd, Hg, etc.) are more soluble and mobile at acid pH. In contrast, elements present in aquatic media as anions (arsenate, vanadate, chromate, etc.) can precipitate or be adsorbed by soil colloids at low pH and mobilise at alkaline pH [40,41,56,57,58,59]. The presence in soil of potentially toxic elements in an easily mobile form causes a deterioration of soil quality and constitutes a threat to the health of soil organisms as well as to human and ecosystem health, in addition to the risk of being exported to the atmosphere, vegetation, and water bodies. After fire, these elements can move vertically or laterally, constituting a risk of contamination of ground and surface waters. They are persistent pollutants prone to bioaccumulation and biomagnification. High concentrations of trace elements interfere with physiological functions of plants and soil microorganisms and inhibit seed germination [7]. Various trace elements and radionuclides have been shown to induce mutations in plants, yeasts, or bacteria [60].

The ecotoxicological risk posed by heavy metals and trace elements has led to the establishment of guidelines to protect human and ecosystem health. Table 4 lists values of the Dutch and Canadian guidelines as well as values of maximum allowable concentrations and trigger action values published by Kabata-Pendias [61] for agricultural soils.

Numerous publications deal with heavy metals and trace elements in ash and burnt soils. Usually, these studies analyse heavy metals in burnt and unburnt soils, although the sampling dates after fire vary widely, from a few days to several years.

Young and Jan [64] collected ash samples during and shortly after a wildfire in California in 1975. The samples were analysed for silver (Ag), cadmium (Cd), chromium (Cr), copper (Cu), iron (Fe), manganese (Mn), nickel (Ni), lead (Pb), and zinc (Zn). The results revealed that the fire mobilised significant amounts of metals, which were deposited with the ash. The metals deposited in highest amounts (4000–50 µg m^−2^) were Fe > Zn > Pb > Mn > Cu, while Ni > Cr > Cd > Ag were deposited in minor quantities (<10 µg m^−2^). These quantities were estimated between 1.8 (for Cd) and 7.8 (for Cr) times higher than the aerial fallout inputs in non-fire conditions. The results were presented on a surface basis and no soil samples were analysed.

Parra et al. [50] determined manganese forms in surface and subsurface samples of a Spanish soil under *Pinus pinaster* Ait., ten months after a wildfire. They reported higher concentrations of total Mn, easily reducible Mn, and Mn associated with organic matter in burnt soils, compared to control soils, in both surface (0–5 cm) and subsurface (5–40 cm) horizons. They attributed, at least partially, the increased Mn concentrations in burnt soils to manganese contributed by the ash. The total Mn concentrations ranged between 470 and 1430 mg/kg in surface horizons and between 300 and 780 mg/kg in subsurface horizons. The Dutch and Canadian laws do not provide threshold values for soil concentrations of manganese, which is an abundant element in the Earth’s crust. The reported Mn concentrations in burnt soils are higher than the world soil average of 488 mg/kg [61] in surface horizons and some subsurface horizons. Adams [65] considers that easily reducible manganese determines the toxicity of this element in soil and sets the phytotoxicity threshold at 50–100 mg/kg. In Parra et al. [50], the easily reducible manganese concentration in unburnt soils was close to 100 mg/kg in surface horizons and clearly lower in subsurface horizons, while in burnt soils the easily reducible manganese exceeded this value both in surface (126–470 mg/kg) and subsurface (5–470 mg/kg) horizons. 

In the case of mercury, a volatile element, most of the soil mercury mobilised by fire is emitted to the atmosphere in gaseous form. Soils act as mercury sinks but, with an increasing incidence of fires, they can become sources of Hg emissions into the atmosphere [66,67]. Global estimates of wildfire-related mercury emissions vary between 100 and more than 1000 Mg yr^−1^ [68]. Volatilised Hg can be transported by the wind and finally transferred to soil or to water bodies. For these reasons, the behaviour of mercury in soils upon burning differs from other trace elements.

Numerous publications study the Hg concentrations in vegetation, litter, ash, and mineral soils, in both burnt and unburnt areas [66,69,70,71,72,73,74,75]. Usually, the mineral soil is the major mercury reservoir, but Hg in leaves, bark, and litter is more reactive and plays a more important role in Hg cycling [69]. Most of the reviewed publications show that Hg concentrations decrease in ash compared to litter (Table 5), the decrease depending on fire intensity and type of vegetation. The Hg concentrations in mineral soil either decreased [66,70,71,72,73,74,75] or did not vary significantly [69,74] upon burning. The decreases in Hg concentration resulted from mercury volatilisation or runoff losses. After the initial loss of Hg in burnt surface soils, the soil Hg concentration increased over time, by mercury sorption from the atmosphere [72,73]. Some studies [66,75] report a decrease of Hg concentration in ash and an increase in the topsoil after rainfall events, indicating the transfer of Hg from ash to soil by rainwater and possibly the fallout of atmospheric Hg. In soils unpolluted prior to fire, the concentrations of Hg in the burnt soils were below the threshold values reported by Kabata-Pendias for agricultural soils (Table 4, [61]). In a study carried out by Abraham et al. [66] on the effects of a prescribed fire in an ancient gold mining site in Australia, heavily contaminated by Hg, the Hg concentrations in soil, both before and after fire, were above the threshold values reported by Kabata-Pendias [61] and, according to the authors, were in most cases below the Australian guidelines. The values of the potential ecological risk index showed that the study area is creating an ecological risk by Hg, particularly after rainfall.

De Marco et al. [76] analysed Mn, Fe, Cu, Pb, and Cd in a shrubland sandy soil in Italy subject to experimental fires of two different burn severities. Total and available Mn, Fe, Cu, Pb, and Cd were determined over a period of three years in burnt and adjacent unburnt soils. Soils (0–5 cm depth) were sampled in the dry and in the wet seasons, at different times after the fire. Soils subject to experimental fire had significantly higher concentrations of all available trace elements and total Mn, Fe, Cu, and Pb, but not significantly different concentrations of total Cd (Table 6). The concentrations of available Fe, Cu, and Pb were lower in soils subject to high severity fire than in soils subject to low severity fire. The concentrations of the toxic Pb and Cd in available form in burnt soils fell, according to the authors, within the normal background range. The total concentrations were below the Dutch and Canadian guidelines and the threshold values reported by Kabata-Pendias [61]. Furthermore, fire returns nutrients held in the above-ground biomass to the soil, promoting the cycling of nutrients and preventing nutrient limitation.

The US Geological Survey analysed ash and burnt soils from various locations in southern California after wildfires in 2007 and 2009 [83,84]. Samples from burnt residential areas had elevated concentrations of As, Pb, Sb, Cu, Zn, and Cr (Table 7). In some cases, the concentrations were above the preliminary remediation goals (PRG) for soils of the US Environmental Protection Agency (USEPA). Moreover, the concentrations of all analysed elements in residential areas were above the Dutch and/or Canadian guidelines. Selected samples with high concentrations of arsenic, selenium, and chromium were analysed to determine the speciation. Water soluble chromium was predominantly Cr(VI). The water extractability of Cr(VI) present in ash is of particular concern because of its toxicity and carcinogenicity. The major arsenic form was generally As(V). Selenium (IV) and (VI) were present at very low levels (<2 ppb in most samples). Ash and soils sampled from burnt residential areas had higher concentrations of arsenic and total and hexavalent chromium than those from wildland areas [83,84]. Maximum trace element concentrations in ash samples from these studies are presented in Table 7 along with the corresponding values of preliminary remediation goals.

Pereira and Úbeda [85] analysed water extractable Al^3+^, Mn^2+^, Fe^2+^, and Zn^2+^ in ash from a wildfire in Portugal. Al^3+^ (12.51 ± 7.01 mg L^−1^) presented the highest and Zn^2+^ (1.25 ± 1.11 mg L^−1^) the lowest concentrations. This is not surprising, since Al, Fe, and Mn are relatively abundant elements in the Earth’s crust, while Zn is usually an element of anthropogenic origin. Al and Mn showed lower spatial variability than Fe and Zn. The concentrations of soluble Al^3+^ and Mn^2+^ (4.25 ± 1.76 mg L^−1^) in the ash were related to the dominant tree species (*Pinus pinaster* or *Quercus suber*), while the concentrations of soluble Fe^2+^ (9.38 ± 9.43 mg L^−1^) and Zn^2+^ related to topography. No data on total concentrations were presented.

In a study carried out in Lower Silesia (Poland), Bogacz et al. [54] determined heavy metals (Zn, Cu, Pb, Ni, Cr) in organic soils affected by fires and compared them with the Polish soil quality standards. The soils were sampled immediately after a fire and 2, 12 and 21 years after a fire. Soils were enriched in Zn, Cu, Cr, Ni and Pb (Table 6), mainly in surface horizons, although the concentrations did not usually exceed the Polish soil quality standards. However, the Dutch and Canadian guidelines, as well as the Kabata-Pendias’ thresholds (Table 4), were exceeded with some frequency for Zn, Cu, Pb, and Ni. The heavy metal concentrations were higher in soils sampled a short time after fire.

Jovanovic et al. [77] determined and carried out fractionation of Cu, Pb, Cd, and Zn in burnt soils from Serbia and compared them with undisturbed soil. The total heavy metal concentrations, except Cd, were higher in burnt relative to unburnt soils in most samples (Table 6). Moreover, burnt soils were enriched in all determined exchangeable metals, easily mobile, and available to plants. The acid-reducible fraction was enriched in Zn and Pb, while the organic fraction was enriched in Cu, Zn, and Pb in burnt soils. The residual fraction, considered as an inert immobile phase, was enriched in Cu, Zn, and Pb in burnt soils. According to the authors, the total concentrations of all analysed metals were close to their average concentration in the Earth’s crust, so that soils could be considered unpolluted. However, only Cu concentrations were below the Dutch and Canadian guidelines and the Kabata-Pendias thresholds (Table 4). Some Cd concentrations, in both unburnt and burnt soils, were above the Canadian environmental quality threshold for agricultural soils. The Pb concentrations in burnt soils were above the Canadian environmental quality threshold for agricultural soils. The Zn concentrations were below the Dutch and Canadian guidelines, but in the case of burnt soils were above some values of maximum allowable concentrations in agricultural soils published by Kabata-Pendias (Table 4).

Kristensen et al. [78] analysed lead in soil and ash samples from three relatively pristine sites in Australia shortly after three wildfires. Soil samples were collected from 0–2 cm and 40–50 cm depths. The 40–50 cm soil samples were used to determine local natural background soil lead concentrations and isotopic composition. Lead concentrations in soils ranged from 4 to 23 mg kg^−1^, while Pb concentrations in ash ranged between 1 to 36 mg kg^−1^. The lead isotopic compositions of most ash and surface soils indicate that the origin of Pb in these samples was a mixture of natural (lithological) lead and historical industrial lead depositions. The concentrations were below the Dutch and Canadian guidelines and the Kabata-Pendias thresholds (Table 4).

In a study conducted in the Marão Mountains (Portugal), Costa et al. [79] analysed ash five months after a wildfire and soil five and eleven months after the wildfire, in an area dominated by *Pinus pinaster* Ait. They reported high concentrations of Mn in the ash, which are attributed to Mn accumulation in pine needles, and estimated the amount of manganese released by the fire at 350 g per hectare. However, the Mn concentrations in both soil and ash (Table 6) were below the world soil average (488 mg kg^−1^, [61]). Five months after fire, the Mn concentrations in soil were higher than in ash, while the Zn and Cu concentrations were lower in soil than in ash. The reported concentrations of Mn and Zn in burnt soils were significantly higher than in unburnt soils five months after the wildfire, but similar to those in unburnt soils eleven months after the wildfire, which is attributed by the authors to high Mn and Zn solubility. These elements can be leached by runoff water and reach water courses, which still had high Mn concentrations one year after the fire, resulting in a loss of water quality. In contrast, the Cu concentrations in soil increased from five to eleven months after the wildfire. The total concentrations of Zn and Cu in soils were always well below the Dutch and Canadian guidelines and the Kabata-Pendias thresholds (Table 4). 

Burton et al. [80] determined trace elements in burnt soils, ash, and ash leachates after a wildfire in California (USA). They reported trace element concentrations in burnt soils not significantly different from the unburnt soil and comparable to concentrations in other unburnt soils from western USA. Moreover, the range of trace element concentrations in ash was similar to other published values and varied by type of ash (white, coloured, or mixed ash). Concentrations of As, Pb, Mn, and Ni were significantly lower in white ash (more completely combusted ash). The concentrations of Cu, Pb, and Zn were higher in ash samples than in burnt soils, while the concentrations of As, Mn, and Ni did not differ significantly between ash and burnt soils. The authors concluded that the wildfire played a minor role in mobilising Cu, Pb, Ni, Se, and Zn during storms, whereas it played a major role in mobilising Mn, Hg, and As. The Mn concentrations (Table 6) were very often above the world soil average (488 mg kg^−1^, [61]) in both burnt and unburnt soils. The Fe concentrations in burnt soils were often above its average concentration in the Earth crust. The concentrations of Cu, Zn, and Cd were always well below the Dutch and Canadian guidelines (Table 4). The concentrations of Ni, Cr, and As were often above the Canadian environmental quality guidelines (Table 4) in both burnt and unburnt soils. The Pb concentrations in some burnt soils were above the Canadian environmental quality threshold for agricultural soils.

Santín et al. [86] determined trace elements (B, Cu, As, Cd, Hg, and Pb) in litter, ash, and soil after a wildfire in a dry sclerophyll eucalypt forest south-west of Sydney, Australia. The sampling was carried out 10 weeks after the fire. Ash production increased significantly with fire severity. Cd and Hg concentrations in ash did not differ significantly among fire severities (Table 8). Hg concentrations in ash were always low and below those in litter and soil, pointing to a loss by volatilisation, in accordance with the findings in above discussed studies. B, As, and Pb concentrations were significantly higher in ash formed under extreme fire severity, indicating a relative enrichment of these elements at the greatest severity due to loss of other elements. In general, the concentrations of these elements in ash were lower than those reported in other studies, in accordance with low concentrations in litter and undisturbed soil. Water soluble elements in ash were relatively low, ranging from <1% for As, Cd, and Pb and <5% for Cu to 6–14% for B. Water soluble As concentration was highest while B, Cu, and Pb concentrations were lowest in extreme fire severity ash, suggesting that different fire severities resulted in different geochemical forms and chemical behaviour of the studied elements. Despite the relatively low concentrations of potentially toxic elements in the ash, there is a risk of contamination of surface waters during a major storm event, given the amount of total ash loads. The concentrations in soils of all analysed potentially toxic elements were always below the Dutch and Canadian guidelines and/or the Kabata-Pendias thresholds (Table 4).

Campos et al. [55], in a study carried out in the Aveiro district (Portugal) after a moderate wildfire that affected an area covered by *Eucalyptus globulus* Labill. and *Pinus pinaster* Ait. plantations, reported significantly higher concentrations of V, Mn, Ni, Cd, and Pb in burnt compared to unburnt soils (Table 6). Sampling was carried out immediately and 4, 8, and 15 months after the fire. The highest increase in burnt relative to unburnt soils was observed for manganese. The increased metal concentrations in burnt soils were related to high concentrations in the ash from burnt vegetation. Over time, the concentrations of Mn and Cd in soil decreased dramatically after the first rainfall events, which is attributed to erosion and leaching losses; in contrast, the concentrations of V, Co, and Ni increased during the first 8 months and Cu and Pb did not change significantly during the study period. These behaviours indicate a higher solubility/mobility and easier desorption of Mn and Cd relative to other heavy metals. This mobility carries a risk of contamination of water bodies. The increased concentrations of V, Co, and Ni in soil four and eight months after fire denote that they are less soluble and mobile in soil than Mn and Cd and persist in the soil, while they are efficiently translated from ash to soil. Cu and Pb persisted in soil in similar concentrations 15 months after fire, accumulating in surface soils, probably in the form of oxides, hydroxides, and carbonates, limiting their leaching to groundwaters. Mn and Pb had the highest concentrations in the ashes. The concentrations of V, Mn, Ni, Cu, and Cd in the ashes declined sharply in the first 4 months, the largest decreases being those of Mn and Cd, and the smallest that of Pb, in agreement with their relative mobilities. Metal concentrations in soils were moderately and positively correlated to pH and to EC in the cases of Mn and Cd and slightly and positively correlated to soil pH in the cases of Pb and V. Soil organic matter correlated significantly and positively to Mn and Cd concentrations and negatively to Co, Cu, and Ni concentrations. The fire-related enrichment factors in soils were close to 1 for Cu and Co and higher than 1.5 for the remaining elements. The maximum enrichment factors were found for Mn (average of 7.6), followed by Cd and Pb (averages of 2.3). The Mn concentrations were below the world soil average [61] in both burnt and unburnt soils. The application of the Dutch and Canadian soil quality standards (Table 4) to assess the soil contamination after fire indicated that some of the reported concentrations (Table 6) were above the Canadian environmental quality threshold for agricultural soils only in the case of Pb. According to the authors, the increased concentrations of trace elements might negatively impact soil functioning through their toxic effects on soil microorganisms. Moreover, they pose a risk of contamination of surface and ground water bodies, within and downstream of a burnt area.

Brito et al. [38] determined major and trace elements in ash and soil samples after three wildfires in the Cerrado region, in Brazil. The studied areas corresponded to three types of vegetation: Cerrado stricto sensu, pasture, and transition area. Ash and soils (0–5 cm) were sampled one day after the end of each wildfire and analysed for total Al, B, Ca, Cd, Cr, Fe, K, Mg, Mn, Mo, P, Pb, S, Si, Sr, Ti, V, Zn, and Ni. Moreover, water-soluble elements were determined in ashes. The three studied areas showed a wide range of element concentrations in soil and ash. The concentrations of B, Ca, K, Mg, Mn, P, S, Si, Sr, and Zn were higher in ash than in the soil, while Al, Cd, Cr, Fe, Mo, Pb, Ti, and V were found at higher concentrations in the soil than in the ashes. The solubilisation rates of different elements in ashes ranged from <0.01% to 26%. The most soluble elements were S, K, and Mg. Among trace elements, the solubilisation rates were highest for Mo and B, and always lower than 0.01% for Cd, Fe, and Pb, in accordance with [86]. The reported concentrations of Cd, Cr, and V in soils were above the Canadian environmental quality threshold for agricultural soils, while the concentrations of Ni, Zn, and Pb were below the Dutch and Canadian guidelines (Table 4).

The mobilisation of ten potentially toxic elements (As, Cd, Co, Cr, Cu, Hg, Mn, Ni, Pb, and Zn) in surface forest soils of a legacy gold mining area in Victoria (Australia) after a controlled burn was studied by Abraham et al. [81,82]. Surface soils (0–3 cm) were sampled two days before the fire, two days after the fire, 3, 6, 9, and 12 months after the fire, and immediately after a major rainfall event. Ash was sampled two days after the fire. The concentrations of potentially toxic elements (PTE) in both unburnt and immediate post-burn soils (Table 6) were in the order Mn > Zn > As > Cr > Cu > Pb > Ni > Co > Hg > Cd. The concentrations of six PTE increased upon burning: Zn (by 87%), Mn (72%), Cd (45%), As (11.5%), Ni (6%) and Co (3.7%). The concentrations of Hg, Pb, Cr, and Cu were lower in the immediate post-burn compared to pre-burn soils, by 27%, 15.4%, 12%, and 2%, respectively. However, only the differences in Zn, Mn and Hg concentrations were significant (*p* < 0.05). Although the concentration of Cu was almost unchanged in surface soil, it showed a high concentration in the ash (145 mg kg^−1^). The increases in PTE concentrations are considered to be due to the accumulation of ash. The decrease in Hg concentration was attributed to volatilisation, volatilisation of Pb is also suggested. Mn (330–2790 mg kg^−1^) and Zn (221–555 mg kg^−1^) had the highest concentrations in ash, whereas Cd (0.02–0.22 mg kg^−1^) and Hg (0.02–0.41 mg kg^−1^) had the lowest concentrations. Concentrations of Cu (56.1 to 207 mg kg^−1^) and As (20.08 to 260 mg kg^−1^) were considerable. The presence of Mn in highest concentrations is in accordance with other studies [45,55]. The concentrations of Mn, Zn, Cu, and Ni in ash were higher than their concentrations in soil, while As, Cd, Co, Cr, Hg, and Pb had higher concentrations in soil than in ash. PTE in ash can be remobilised through post-fire water runoff and wind. From two days after the burn, the concentrations of Mn and Zn decreased during the study period to pre-burn levels. Cd, Ni, and Co showed a similar behaviour. Arsenic showed an increase immediately after the burn, decreases 3 and 6 months after the burn, and a further increase 9 months after the burn. The concentrations of Cr and Pb decreased immediately after the burn and increased 3 months after the burn; in the following sampling period, there was reduction or no change. An intense rainfall event 13 months after the burn caused considerable runoff and leaching, resulting in decreased concentrations of most PTE. The concentration of Mn in burnt soils reaches values considerably higher than the world soil average. The concentration of Cu in burnt soils exceeds the Canadian guidelines values in some samples. Zn concentration is often above the Canadian guidelines while As always exceeds these values in both burnt and unburnt soils (Table 6).

Harper et al. [39] determined Al, B, Cu, Fe, Ni, As, Cd, Hg, and Pb in ash from wildfires in Australia, USA, Canada, Spain, and the UK, sampled after the wildfire and before any rainfall. Al and Fe, which are lithogenic elements, showed high concentrations in ash (1320–22,600 and 979–30,600 mg kg^−^^1^, respectively). The highly toxic As (0.46–9.67 mg kg^−^^1^), Cd (0.17–1.13 mg kg^−^^1^), and Hg (0–0.05 mg kg^−^^1^) presented the lowest concentrations, which were below the Dutch and Canadian threshold values for soils (Table 4). The variations in concentrations may be explained by the accumulative capacity of the different vegetation types, fire temperature, and soil properties. The concentrations of water-soluble trace elements were Cd, 0–7 µg kg^−^^1^; Ni, 60–844 µg kg^−^^1^; Zn, 0–140 µg kg^−^^1^; and Hg, 1–2 µg kg^−^^1^. On average, the proportions of water-soluble Al, Pb, Mn, Fe, and Zn were less than 1%; As, Ni, Cu, Cd were less than 5%; and Hg ranged between 5 and 57%.

Alexakis [45] studied the concentrations of trace elements in ash from a wildfire in Greece, affecting wildland and residential areas. Ash was sampled at 27 sites in wildland (6 sites) and residential (21 sites) areas two months after the fire and analysed for total Ag, Al, As, B, Ba, Be, Bi, Ca, Cd, Co, Cr, Cu, Fe, Ga, Hg, K, La, Mg, Mn, Mo, Na, Ni, P, Pb, S, Sb, Sc, Sr, Th, Ti, Tl, U, V, W, and Zn. Trace element concentrations were compared with soil quality guidelines and threshold values established for ecological and human health risk. Moreover, the elements in ash that pose a health risk for human and terrestrial organisms were identified. The median values of trace elements in wildland ash decreased in the order: Mn > Ni > Zn > Ba > Sr > Cr > Pb > B > V > Cu > Co > As > La > Sc > Sb > Be > Cd. In residential ash, the order was: Mn > Zn > Sr > Ba > Ni > Pb > B > Cr > Cu > V > La > As > Co > Sc > Sb > Cd > Be. The median concentrations of B, Ba, Cd, Cu, P, Pb, Sr, and Zn were higher in residential ash than in wildland ash, whereas the median concentrations of As, Be, Co, Cr, Mn, Ni, and Sc were higher in wildland ash than in residential ash. The major sources of Pb in residential ash are the former use of leaded gasoline, the use of Pb-based paints, and old lead pipes. According to the authors, the concentrations of Al, As, Co, Fe, Mn, Ni, Sb, and Zn in the wildfire ash pose a potential risk to human health. As, B, Ba, Cu, Mn, Ni, Pb, Sb, V, and Zn may pose a threat to plant, reptile, and mammal species. 

Alexakis et al. [87] determined bioavailable forms of copper (Cu), iron (Fe), manganese (Mn), and zinc (Zn) in topsoils (0–5 cm depth) and subsoils (5–25 cm depth) from burnt and unburnt sites two months after the same wildfire event in Greece as in the previous reference. The concentrations of bioavailable Fe and Mn were significantly higher in burnt compared to unburnt topsoils. This higher availability is attributed to the addition of ash during the wildfire event.

The reviewed publications show that the mobilisation of trace elements by fire and the associated risks are low in unpolluted areas and more important in areas with high pre-fire concentrations of these elements in soils or vegetation. The concentrations of trace elements in the ash depend on the vegetation from which it originates and, in the case of fires that affect urban areas or human settlements, on the presence of these elements in houses or infrastructures. The mobilisation of trace elements varies with fire severity and poses a risk to atmosphere and water bodies. The burnt soils may act as sources of toxic elements for months or even a few years after fire.

Most published studies on trace elements mobilisation upon burning deal with mobilisation from soil organic matter, litter, or plant tissues. However, fire can also mobilise trace elements associated with soil inorganic compounds. This is the case of arsenic, which in soils is commonly bound to iron oxide minerals, such as ferrihydrite (Fe_10_O_14_(OH)_2_) and goethite (α−FeOOH), mainly as As(V). Arsenate forms inner-sphere complexes with ferrihydrite, goethite, amorphous Fe hydroxides, and Al oxides [88,89]. Johnston et al. [52,53,90] studied the release of arsenic by thermal transformation of As(V)-bearing Fe(III) minerals in soils. Heating to more than 400 °C caused transformation of schwertmannite (Fe_8_O_8_(OH)_6_SO_4_) to nanocrystalline hematite (α−Fe_2_O_3_), which has a smaller particle size and greater surface area. Higher temperatures also caused the As to become progressively more exchangeable, thereby inducing enhanced As mobilisation. In the presence of an organic-rich soil, schwertmannite was transformed to maghemite (γ−Fe_2_O_3_) and hematite at temperatures above 300–400 °C, while As(V) was reduced to As(III). As(III) species are more readily desorbed than As(V) from adsorbing surfaces. Reducing compounds derived from pyrolysis of organic matter are critical to reduce Fe(III) to Fe(II) and As(V) to As(III), enhancing mobilisation of As(III). The results indicate that in acid sulphate soils, where As commonly associates with schwertmannite, moderate fires may generate labile As(III) species and cause As(III) mobilisation. In oxic soils, As(V) associates with oxides such as ferrihydrite and goethite. Heating at temperatures higher than 400 °C transformed ferrihydrite and goethite to maghemite. Moreover, during heating of organic-rich soils, ferrihydrite and goethite-bound As(V) can be rapidly reduced to As(III). Moreover, other authors, such as Ketterings et al. [91], reported the transformation of soil goethite into maghemite upon burning and the importance organic matter for the complete conversion of goethite. Maghemite has a higher crystallinity than ferrihydrite and, therefore, is a less efficient sorbent for both As(III) and As(V). Therefore, moderate-temperature fires of short duration in oxic soils may generate labile As(III) species and lead to As(III) mobilisation. Furthermore, As(III) is more toxic and mobile than As(V) [41,43], posing a higher risk to soils and water bodies.

In a similar way, Burton et al. [92] studied the formation of hexavalent chromium by heating Cr(III)-substituted ferrihydrite, goethite, and hematite or a natural soil rich in hematite, goethite, and ferrihydrite, with a total Cr concentration of 220 mg kg^−^^1^ and undetectable Cr(VI); there is no information on the organic matter content in the soil. Chromium(III) often exists in soil as a structural substituent for Fe(III) in Fe(III) oxide minerals. The authors heated the samples at 200, 400, 600, and 800 °C. By heating Cr(III)-substituted ferrihydrite and hematite, up to ~50% of the initial Cr(III) was oxidised to Cr(VI), the Cr(VI) formation being highest at 200–400 °C. Heating Cr(III)-substituted goethite led to oxidation of up to ~100% of Cr(III) to Cr(VI) as the temperature approached 800 °C. Heating the soil at ≥400 °C also resulted in large amounts of Cr(VI), with a maximum total Cr(VI) concentration of 77 mg kg^−^^1^ (~35% of the soil’s total Cr concentration) at 600 °C. This value is very close to the Dutch intervention value and considerably higher than the Canadian reference values for agricultural and residential uses (Table 4). A relatively large proportion (31–42%) of chromium (VI) is in exchangeable form and, therefore, bioavailable and easily mobile. Cr(VI) forms quickly by oxidation of Fe oxide-bound Cr(III) at temperatures reached in surface soils during fires, so that oxidation of Cr(III) during wildfires may represent a significant source of Cr(VI) in surface soils. Cr(VI) is considerably more mobile and toxic than C(III); so this oxidation may pose a significant environmental risk.

## 4. Pyrolytic Production of Toxic Compounds

The transformation of biomass and soil organic matter at high temperatures can lead to the formation of toxic compounds, especially polycyclic aromatic hydrocarbons (PAH). PAH can occur in pyrogenic materials such as charcoal, ash, and smoke. The presence of these substances in ash and charcoal can contribute importantly to the toxicity of soils and water bodies [93,94,95]. Many PAH have toxic or mutagenic effects on plants [60,96,97], soil insects [98], annelids [99,100], crustacea [101,102], collembola [100], and microorganisms [103]. The concern related to PAH lies mainly in its carcinogenicity to humans, mutagenicity, persistence, and bioaccumulation. Polychlorinated biphenyls (PCB), polychlorinated dibenzo-p-dioxins (PCDD), and polychlorinated dibenzofurans (PCDF) were also reported to be formed or remobilised as a result of fire and are a cause for concern due to their potential effects on humans and wildlife [104,105].

The risks posed by PAH and other toxic organic pollutants to human health and ecosystems had led to the establishment of different guidelines. Table 9 lists values of the Dutch and Canadian soil quality guidelines for PAH and PCDD/F.

According to Freeman and Cattell [106], wood burning can generate high concentrations of PAH. These authors studied the production of PAH from burning wood and other vegetation in Sydney, Australia. They determined by HPLC eleven PAH (fluoranthene, pyrene, benzo[*a*]anthracene, chrysene, benzo[*b*]fluoranthene, benzo[*k*]fluoranthene, benzo[*a*]pyrene, dibenzo[*ah*]anthracene, benzo[*ghi*]perylene, indeno[*cd*]pyrene, and coronene) in airborne particulate matter from various types of combustion (a domestic open wood fire, burning leaves and wood on an open fire, an open barbecue burning wood, a bonfire burning old vegetation and some cardboard, a large-scale uncontrolled bush fire, burning paper and cardboard in a commercial incinerator, and cigarette smoke). Bush fires were the highest sources of chrysene, benzo[*a*]pyrene, and coronene. Though the samples for the bush fire were taken near a road, the high concentrations of coronene cannot be explained by emissions from motor vehicles. According to these authors, burning of vegetation is estimated to provide between 6% and 24% of the PAH, the remainder being attributable to motor vehicles. Bush fires could contribute significantly to the exposure of the population to PAH, in particular to the highly carcinogenic benzo[*a*]pyrene. Particle-bound PAH can be transported long distances in the atmosphere and eventually reach the soil. The concentrations of fluoranthene, pyrene, benzo[*a*]anthracene, benzo[*b*]fluoranthene, benzo[*k*]fluoranthene, benzo[*a*]pyrene, dibenzo[*ah*]anthracene, and indeno[*cd*]pyrene in airborne particulate matter from bush fire (Table 10) exceeded the Canadian soil quality guidelines for environmental health (Table 9).

González-Vila et al. [107] determined, by gas chromatography-mass spectrometry (GC-MS), PAH in different soils that underwent fires. The samples included pine forest soils before, immediately after and two years after a wildfire, soils where an open bonfire of pine trees took place recently or 50 years before, soils that covered home-made pine charcoal kilns, as well as charcoal itself. The highest total concentrations of PAH were found in soils that covered charcoal kilns, followed by soils where an open bonfire took place recently and forest soils immediately after a wildfire. The major determined PAH were naphthalene and phenanthrene derivatives, more condensed PAH being present in minor amounts. Naphthalene derivatives, which are rather volatile compounds, were found almost only in soils covering ovens for charcoal production. Phenanthrene derivatives were abundant in most samples. Fluorene-type PAH were detected in soils immediately after fire. Tetra- and pentacyclic PAH, particularly pyrene and chrysene, were detected in minor quantities. The highly carcinogenic benzo[*a*]pyrene was undetectable. The total accumulation of PAH was at most 2–4 g/ha, much less than those frequently derived from fossil fuel combustion in urban or industrial areas, and do not pose an environmental concern. Moreover, the analysis of samples 2 or 50 years after fire indicated a low persistence of PAH in soils. The concentrations in burnt surface soils (Table 11) were low compared to environmental guidelines (Table 9) and had largely disappeared two years after fire.

Bin Abas et al. [108] sampled smoke particulate matter from a controlled burn of forest litter in Amazonia, Brazil, for analysis by gas chromatography (GC) or GC-MS. Polycyclic aromatic hydrocarbons and oxy-PAH, derived from pyrolysis of biomass, were found at high levels. The results were presented on an air volume basis. The PAH consisted primarily of phenanthrene, methylenephenanthrene, methylphenanthrenes, fluoranthene, pyrene, chrysene, methylchrysenes, and benzofluoranthenes. Benzo[*a*]pyrene and benzo[*e*]pyrene were found at trace level. The smoke may propagate over long distances, spreading PAH over large areas. Pyrogenic organic compounds can disperse over soils and oceans.

Bundt et al. [104] determined 20 PAH and 14 PCB in wood ash intended to be used as a forest fertiliser and in a Swiss forest soil before and after ash application. According to the authors, the results revealed moderate soil pollution by PAH (total concentration 815–1640 µg kg^−^^1^ in the organic layer) and high concentrations of PCB (total concentration 21.7–48.8 µg kg^−^^1^ in the organic layer) before the ash application (Table 12); however, these values are below the Dutch and Canadian guidelines (Table 9). The ash had high concentrations of PAH (total concentration 16.8 mg kg^−^^1^, below the Dutch intervention value of 40 mg kg^−^^1^) and low concentrations of PCB (total concentration 3.4 µg kg^−^^1^). The ash application increased the PAH concentrations in the organic horizons up to sixfold and mobilised PCB stored in the soil (Table 12). This mobilisation is considered to be caused by the high pH of the ash, which brought about the mobilisation of soil organic matter, which in turn acted as a carrier for the organic pollutants. Even so, the concentrations were below the Dutch and Canadian guidelines.

Kim et al. [105] measured PAH and PCDD/F in soil (0–5 cm) and ash samples after forest fires in South Korea. Samples were analysed 1, 5, and 9 months after fire by GC-MS. The concentrations of PCDD/F in the burnt soils ranged from 0.037 to 0.370 ng/g whereas the PAH concentrations ranged from 153 to 1570 ng/g. The concentrations of PCDD/F in burnt soils were higher than in the corresponding unburnt soils one month after fire, but similar to unburnt soils 5 or 9 months after fire. The concentration of PCDD/F (TEQ) in burnt soils was above the Canadian environmental quality guideline (Table 9) in one sample one month after fire (Table 13). The concentrations of PAH in the burnt soils were higher than in the unburnt soils 1, 5, and 9 months after fire (Table 13) but below the Dutch intervention value (Table 9). The PAH and PCDD/F, formed by the combustion of wood and other organic matter during forest fires, are adsorbed onto ash particles and deposited onto the surface soil. The concentrations of PAH and PCDD/F declined over time due to the loss of ash by wind and rain erosion. The PCDD/F and PAH lost from the soils may be transported with ash to the atmosphere or hydrosphere, causing an increase in the concentrations of these compounds in neighbouring environments. In a later study [109], Kim et al. focussed on PAH, reporting the levels of 16 individual PAH. The total PAH concentrations in burnt soils were 4–24 times higher than in unburnt soils. The total PAH levels in the burnt soils one month after fire (average 1200 ng/g) were comparable to those of urban soils but approached with time the values reported for other forest soils. The concentrations of low molecular weight PAH (2–4 rings) in ash and soils were much higher than those of high molecular weight PAH (5–6 rings). Phenanthrene was the most abundant PAH in unburnt soils (Table 13). One month after fire, naphthalene and phenanthrene were the prevailing PAH in the burnt soils. The concentrations of naphthalene decreased and those of phenanthrene increased 5 and 9 months after fire. Low molecular weight PAH (particularly naphthalene) are more easily lost by degradation or volatilisation than high molecular weight PAH. The concentrations of naphthalene and phenanthrene (Table 13) were above the Canadian environmental quality guidelines (Table 9) one month after fire and in some samples, 5 and 9 months after fire. The concentration of pyrene was above the Canadian environmental quality guideline for agricultural use in one sample one month after fire.

Meharg and Killham [110] showed that the domestic burning of coastal peat was a significant source of dioxins and furans. In many coastal communities of Scotland, the ash from this burning was added over centuries to arable soils to improve their fertility, resulting in significant concentrations of dioxins and furans in soil. The authors report concentrations of total dioxins of 114 ng kg^−1^ in unburnt peat, 643 ng kg^−1^ in burnt peat, and 217 ng kg^−1^ in arable soil, which are very high values compared to reference concentrations (Table 9).

García-Falcón et al. [111] monitored for ten months eight representative PAH in the 1–5 cm layer of a burnt peri-urban woodland soil in NW Spain and compared their concentrations with those in a nearby unburnt peri-urban woodland soil, a distant (5 km) unburnt peri-urban woodland soil, and a distant (20 km) unburnt rural woodland soil. The PAH (fluoranthene, benzo[*b*]fluoranthene, benzo[*k*]fluoranthene, benzo[*a*]pyrene, benzo[*ghi*]perylene, indeno[1,2,3-*cd*]pyrene, benzo[*a*]anthracene, and dibenzo[*ah*]anthracene) were determined by HPLC. Moreover, the PAH adsorption capacity of ash was determined. The total concentration of PAH was 26 µg kg^−^^1^ in the unburnt rural soil, which furthermore did not present detectable quantities of the highest molecular weight PAHs, typical of vehicle traffic and other urban sources. Ten days after the wildfire, the PAH concentrations in the burnt soil were very similar to those in the distant unburnt peri-urban soil, their total concentration being nearly seven times that of the unburnt rural soil, and contained significant amounts of high molecular weight PAH. The relatively low PAH concentrations in the burnt soil suggest their retention by the ash layer, which had a very high PAH adsorption capacity (1169 µg kg^−^^1^). Ten months after the wildfire, the total concentration of PAH at the burnt site had declined from 188 to 119 µg kg^−^^1^. At that time, the total PAH concentration in the nearby unburnt peri-urban woodland soil, nearly 500 m downwind from the burnt area, was 791 µg kg^−^^1^, roughly 5 times greater than in the distant peri-urban site. This fact is interpreted as being a result of the transportation of PAH in the fire smoke and their incorporation into the soil organic layer. The total PAH concentrations in the burnt soil (119–209 µg kg^−^^1^) and in the unburnt nearby peri-urban soil were below the Dutch intervention value (Table 9). The concentrations of all the analysed individual PAH were below the Canadian environmental quality guidelines in the burnt soil. The concentrations of benzo[*b*]fluoranthene and indeno[1,2,3-*cd*]pyrene were above the Canadian environmental quality guidelines for agricultural use in the unburnt nearby peri-urban soil.

Mohd Tahir et al. [112] determined 16 EPA-priority PAH [113] in roadside soils affected by recurrent grassland fires in Malaysia. Surface soils (0–10 cm depth) from burnt and unburnt sites were analysed by GC-MS. The total PAH concentrations ranged from 30 to 450 μg kg^−1^, well below the Dutch intervention value, and were not significantly different in burnt and unburnt soils. The PAH profiles were also not significantly different in burnt and unburnt soils. Both biomass burning and vehicle emissions were sources of PAHs in these soils. The highly carcinogenic benzo[*a*]pyrene was detected in almost all sampling stations. The benzo[*a*]pyrene concentrations were below 15 μg kg^−1^ in most samples but reached 80 μg kg^−1^ in one sample.

De la Rosa et al. [114], studying the organic matter in burnt soils, found that PAH were abundant and diverse in burnt soils and possibly come from biomass burning.

Vergnoux et al. [115] analysed 14 priority PAH in burnt forest soils in the south of France. Sampling was carried out one and three years after the wildfire. The study showed the contribution of forest fires to the contents of PAH in surface soils. Moreover, the PAH concentrations decreased with the time elapsed since the last fire, approaching control values in old burnt soils, indicating that a natural remediation takes place after the fire event. However, this soil remediation can occur at the expense of pollution of watercourses. In accordance with the results of Kim et al. [109], the PAH prevailing in these burnt soils were low molecular weight PAH (naphthalene, acenaphtene, fluorene, phenanthrene, anthracene, fluoranthene, and pyrene), while the concentrations of the high molecular weight PAH were not significantly different from control soils. The total PAH concentrations in burnt soils were below the Dutch intervention value. Of the individual PAH analysed, only naphthalene (70 μg kg^−1^) exceeded the Canadian environmental quality guideline.

Sojinu et al. [116] analysed 16 EPA priority PAH, in fire-affected surface soils from a mangrove forest and a nearby unburnt soil in Nigeria, by GC-MS. The total PAH concentration was 19 µg kg^−^^1^ in the unburnt soil and ranged from 63 to 188 µg kg^−^^1^ in burnt soils, well below the Dutch intervention value. According to the authors, the total PAH concentration exceeded the range reported for uncontaminated soils in one of three sites. Naphthalene, fluoranthene, and benzo[*b*]fluoranthene were the major PAH in the studied soils. All the individual PAH were present at higher concentration in the burnt soils than in the control (unburnt) soil. The PAH profiles indicated the grass and wood combustion as a likely source of PAH in these soils. Comparing the measured concentrations with soil quality guidelines, the authors concluded that the PAH in the studied soils did not pose a serious risk to human health. However, naphthalene exceeded the Canadian environmental quality guideline in one soil. In a recent paper, Faboya et al. [117] investigated by GC-MS the concentrations and profiles of PAH in soils from a tropical rainforest in Nigeria affected for decades by recurrent wildfires. Mineral soil (0–15 cm depth) was sampled at four different sites (including a control site) and analysed for 29 PAH. It is worth mentioning that the number of individual PAH analysed is the highest of all the studies reviewed. The total PAH concentration was 136 ng g^−1^ in the control soils and ranged from 104 to 1869 ng g^−1^ (average 713 ng g^−1^), well below the Dutch intervention value, in the burnt soils. The moderately high concentrations of PAH were attributed to the recurrent burning activity. Two- and three-ring PAH prevailed in burnt soils, the most abundant being phenanthrene (average concentration 172 ng g^−1^) and anthracene (average concentration 165 ng g^−1^). One of the soils analysed exceeded the Canadian environmental quality guidelines (Table 9) for phenanthrene (with 487 ng g^−1^) and pyrene (with 216 ng g^−1^). In control soils, phenanthrene and anthracene accounted for 57% of total PAH, with concentrations of 39 ng g^−1^ each. The PAH profiles are consistent with an origin in the combustion of wood and biomass.

Pontevedra-Pombal et al. [118] studied the PAH accumulation over the past 1000 years in an undisturbed ombrotrophic peatland located in a remote rural area in Galicia, NW Spain. The analysed PAH were fluoranthene, pyrene, benzo[*a*]anthracene, chrysene, benzo[*b*]fluoranthene, benzo[*k*]fluoranthene, benzo[*a*]pyrene, dibenzo[*ah*]anthracene, benzo[*ghi*]perylene, and indeno[1,2,3-*cd*]pyrene. The total concentrations of PAH ranged from 16 to 192 µg kg^−^^1^, higher than those measured in rural soils of Galicia. The PAH determined at highest concentrations were fluoranthene, pyrene, indeno[1,2,3-*cd*]pyrene, benzo[*b*]fluoranthene, and benzo[*ghi*]perylene. The concentrations of benzo[*a*]pyrene ranged between 0.1 and 9.4 µg kg^−^^1^. All the analysed individual PAH were below the Canadian environmental quality guidelines. PAH were present in the whole peat profile analysed, to a depth of 40 cm, indicating continuous pollution by PAH in the study area since at least 1050 A.D. The maximum PAH concentrations were measured in the upper 10 cm (after ca. 1700 AD). A secondary concentration peak was found at 38 cm for low molecular weight PAH. Low molecular weight PAH (fluoranthene and pyrene) prevailed in the period 1050–1600, while after 1600 or 1700, high molecular weight PAH, related to urbanisation/industrialisation, increased significantly. Based on the relative concentrations of various PAH, the authors consider that PAH in the studied site mainly derived from the combustion of coal, wood, and vegetation, and relate the accumulation of low molecular weight PAH during 12th–13th centuries with the use of fire for the expansion of agricultural land.

Campos et al. [94] tested the toxicity to four aquatic species of runoff from a burnt eucalypt stand in Portugal, collected immediately after the wildfire and nearly one year later. The two runoff samples differed in PAH concentration (higher in the first runoff) and composition. Both runoff samples produced significant inhibitory effects on the three species representing the lower trophic levels, i.e., the bacteria *Vibrio fischeri*, the alga *Pseudokirchneriella subcapitata*, and the macrophyte *Lemna minor*, but not on the invertebrate *Daphnia magna*. Unexpectedly, the runoff collected one year after fire was the most toxic to *V. fischeri*, *P. subcapitata*, and *L. minor*, possibly due to the predominance of naphthalene in this sample. Likewise, Silva et al. [95] prepared aqueous extracts of wildfire ash, analysed them for PAH and tested their toxicity to *V. fischeri*, *P. subcapitata*, *L. minor*, and *Daphnia magna*. Only two low molecular weight PAH (naphthalene and phenanthrene) were present in quantifiable amounts in the extracts. The ecotoxicological assays revealed that the extracts, like runoff waters, induced a significant decrease in the growth of *P. subcapitata* and *L. minor* and inhibited the luminescence of the bacterium *V. fischeri*, without any significant effect in *D. magna*. In a later article [93], Campos et al. studied the influence of forest fires in PAH concentrations and profiles in ash and topsoils in *Pinus pinaster* Ait. and *Eucalyptus globulus* Labill. stands in Portugal. Fifteen US EPA priority PAH were analysed. The total PAH concentrations changed from 34–53 ng g^−1^ in an unburnt eucalypt soil, typical for uncontaminated soils, to 132–242 ng g^−1^ immediately after fire. The PAH concentrations were considerably higher in ash (315–695 ng g^−1^) than in topsoil. In both ash and topsoil, the PAH concentrations were higher in pine than in eucalypt stands. In both ash and topsoil, the PAH concentrations decreased significantly with time after fire. From four months after fire onwards, the PAH concentrations in eucalypt soils were no longer significantly different from those in a nearby unburnt soil. This decrease was attributed to export by runoff or leaching, with the consequent risk of contamination of water bodies, as well as to degradation. The fire severity did not influence the total concentrations of PAH in the burnt soils or in the ash. Consistent with [109] and [115], the wildfire changed the PAH composition, with enrichment in three-ring PAH (69% vs. 27% of total PAH immediately after the fire in burnt and unburnt eucalypt soils, respectively). The three-ring PAH were always predominant in the ash, especially in the case of the less severe fire. The PAH profiles point to plant biomass combustion as the source of the immediate postfire PAH. Immediately after fire, the relative fraction of the three-ring PAH in soil was higher in eucalypt than in pine stands and decreased with the time elapsed after fire. The preferential disappearance of the three-ring PAH is consistent with their greatest water solubility and volatility.

Choi [119] determined by GC/MS 16 US-EPA priority PAH in pine bark, litter, and surface soil samples collected one, three, five, and seven months after a forest fire in South Korea. Maximum total PAH concentrations were determined one month after fire, with average values of 5920 ng g^−1^ in pine bark, 1540 ng g^−1^ in litter, and 133 ng g^−1^ in soil, while in control (unburnt) samples the concentrations were 124, 75, and 26 ng g^−1^ in pine bark, litter, and soil, respectively. Thereafter, the PAH concentrations progressively decreased over time, but remained above the control values until seven months after fire (last sampling). The decrease was attributed to leaching, volatilisation, and degradation and was slower in soils than in bark and litter. The low molecular weight (2–4 rings) PAH prevailed over the high molecular weight (5–6 rings) PAH, especially in bark, showing that more directly burnt samples have relatively higher light PAH/heavy PAH ratios. Naphthalene (2 rings) was the prevalent PAH in bark and litter (76% and 44% of the total PAH, respectively), followed by phenanthrene (3 rings), fluoranthene, pyrene, and chrysene (4 rings). The soil samples also showed relatively high fractions of naphthalene and phenanthrene. The ratios fluoranthene/(fluoranthene + pyrene) were consistent with an origin in biomass burning. In a later paper [120], Simon et al. monitored the same 16 PAH in burnt and unburnt soils (0–5 cm depth) and ash for 16 months after a forest fire in South Korea. The total PAH concentrations ranged between 120 and 335 ng g^−1^ in the control soils, pointing to moderate contamination and representing the semi-rural characteristics of the study area, and decreased with time after fire. Tetracyclic PAH were dominant, averaging 41% of total PAH, while phenanthrene (15%) was the most abundant individual compound. In burnt soils, the total PAH concentrations decreased from 294 ng g^−1^ (19 days after fire) to 40 ng g^−1^ (492 days after fire), in accordance with [119]. Although tetracyclic PAH were dominant in burnt soils, they were enriched in light (2–4 ring) PAH compared to control soil, naphthalene (22%) and phenanthrene (19%) being the most abundant individual compounds. The total PAH concentrations in the ash samples decreased continually from 11,007 ng g^−1^ (19 days after fire) to 1169 ng g^−1^ (492 days after fire). The ash samples were also enriched in light PAH. The decreasing trend of total PAH in ash was driven by light PAH, indicating that low molecular weight PAH were dominantly produced by the forest fire. The PAH profiles indicated an origin in biomass burning. The PAH leached from the ash layer, particularly the water-soluble light PAH, are mostly transported by surface runoff and, to a much lesser extent, transferred to the underlying soil. Thus, the rainfall events occurred after the fire lead to removal of PAH from burnt trees and ash layer and consequent contamination of surface waters.

Mansilha et al. [121] analysed for 16 PAH spring water samples from burnt and unburnt areas in Serra do Gerês and Serra da Estrela, Portugal, with the aim of investigating the impact of extensive forest fires on groundwater contamination by PAH. The presence of PAH in groundwater would reveal the inefficiency of the soil as a filter for these pollutants. All analysed PAH, except the highly carcinogenic benzo[*a*]pyrene, were found in samples analysed in burnt areas, the total concentrations ranging from 23.1 to 95.1 ng L^−1^. Mostly the PAH concentrations in burnt areas were significantly higher than in control (unburnt) areas (average total concentration 16.2 ng L^−1^). Naphthalene, fluorene, anthracene, benzo[*a*]anthracene, dibenzo[*ah*]anthracene, benzo[*ghi*]perylene, and indeno[1,2,3-*cd*]pyrene were the main PAH resulting from burning. In control samples, the low molecular weight PAH prevailed, naphthalene accounting for 60% of the total concentrations. In burnt area samples, naphthalene contributed 41% and 5- and 6-rings PAH, not present in control samples, were found in significant amounts, dibenzo[*ah*]anthracene, benzo[*ghi*]perylene and indeno[1,2,3-*cd*]pyrene contributing 27% of the total concentration.

Marynowski et al. [122], analysing paleo-charcoal samples, a proxy for ancient wildfires, found adsorbed PAH at relatively low concentrations. According to the authors, their distribution, with a significant contribution from typical pyrolytic compounds such as anthracene, 4H-cyclopenta[*def*]phenanthrene, benzo[*a*]anthracene, and benzo[*a*]pyrene, was typical for rapid combustion.

Dymov et al. [123,124] analysed 15 PAH (naphthalene, acenaphthene, fluorene, phenanthrene, anthracene, fluoranthene, pyrene, benzo[*a*]anthracene, chrysene, benzo[*b*]fluoranthene, benzo[*k*]fluoranthene, benzo[*a*]pyrene, dibenzo[*ah*]anthracene, benzo[*ghi*]perylene, and indeno[1,2,3-*cd*]pyrene) in burnt and unburnt podzol soils from the Russian taiga. The unburnt soil presented moderately high contents of PAH in the lower subhorizon of the litter (total concentration 432 ng g^−1^), particularly chrysene, phenanthrene, naphthalene, and fluorene, which are thought to be associated with former fires evidenced by the presence of charcoal in the upper mineral horizons. The PAH concentrations in the mineral horizons were much lower, with a predominance of the relatively soluble fluorene and phenanthrene. In the burnt soil, the concentrations of all PAH increased notably in the pyrogenic litter horizon, rich in charcoal particles, the total concentration reaching 1910 ng g^−1^. The PAH concentrations were notably higher two years after fire than 10 or 16 years after fire, but even 16 years after fire, the concentrations were significantly higher than in control (unburnt) soil. The concentrations of chrysene, fluorene, naphthalene, anthracene, and pyrene increased most significantly. The total concentration of PAH slightly increased after the fire in the lower organic subhorizon, fluorene and naphthalene increasing the most. An increase in the total concentration of PAH was also observed in the illuvial Bh horizon, which could be due to migration in water.

Tsibart et al. [125] studied the soil contamination by PAH in drained peatlands affected by smouldering wildfires in the Moscow region, Russia. In peat smouldering, the fire propagates slowly and deep soil horizons are affected by high temperature; these conditions favour PAH formation. The burning gave rise to new ash horizons, charry peat horizons, and incipient O horizons. The PAH naphthalene, phenanthrene, chrysene, pyrene, anthracene, benzo[*a*]anthracene, benzo[*a*]pyrene, benzo[*ghi*]perylene, fluorene, dibenzotiophene, triphenylene, benzo[*e*]pyrene, benzo[*k*]fluoranthene, and coronene were determined by spectrofluorometry in various horizons of the studied soils. The total concentrations of PAH ranged from 0.2 to 137.2 ng g^−1^ in unburnt soil samples and from 0.0 to 332.6 ng g^−1^ in burnt soil samples. The highest PAH concentrations were found in charry peat horizons and in post-fire incipient O horizons. Naphthalene, phenanthrene, and benzo[*a*]anthracene were the major PAH in unburnt soils. Besides low molecular weight PAH, 5–6 rings PAH (benzo[*ghi*]perylene, benzo[*e*]pyrene, and benzo[*k*]fluoranthene) appeared in charry peat horizons. Benzo[*a*]pyrene concentrations never exceeded 3 ng g^−1^. The measured PAH concentrations were not hazardous for biota or humans. The accumulation or not of PAH in burnt peat soils depended on the initial thickness of the peat horizon [126].

Chen et al. [127] focussed on the effects of burn intensity on the PAH produced by wildfires. They determined 16 EPA-regulated PAH, 6 chlorinated PAH, and 3 brominated PAH in forest soils affected by severe or moderate wildfires and unburnt soils in California, USA. Ash + soil samples (0–5 cm) from severely burnt, moderately burnt, and unburnt sites were collected and analysed. Contrary to [93], the fire severity significantly influenced the total PAH concentration. The total PAH concentration in moderately burnt ash + soil (893 µg kg^−^^1^) was significantly higher than those in unburnt soil (247 µg kg^−^^1^) and in severely burnt ash + soil (515 µg kg^−^^1^). The relatively high PAH concentration in the unburnt soil is attributed to previous wildfires. The total concentrations of chlorinated PAH were 3.58 µg kg^−^^1^ in unburnt soils, 3.63 µg kg^−^^1^ in moderately burnt samples, and 1.03 µg kg^−^^1^ in severely burnt samples. Thus, moderate burning had no effect but severe burning decreased the chlorinated PAH concentrations. In contrast, the total concentrations of brominated PAH were significantly lower in both moderately burnt (9.26 µg kg^−^^1^) and severely burnt (2.66 µg kg^−^^1^) samples compared to unburnt soils (16.33 µg kg^−^^1^). Therefore, wildfires are not a significant source of soil chlorinated or brominated PAH, rather severe fires cause the loss of halogenated PAH from soil. Consistent with other studies, the wildfire modified the PAH profiles, although the PAH were in all samples dominated by naphthalene, followed by phenanthrene. The percentage of naphthalene in unburnt soils was significantly higher (72.8%) than in moderately burnt samples (43.9%) but did not differ significantly from severely burnt samples (57.4%). The percentage of phenanthrene was lower in unburnt soils compared to burnt samples. The PAH concentrations in both burnt and unburnt samples were dominated by low molecular weight (<4 rings) PAH, which accounted for 94–97% of total PAH. According with the variations in concentrations, a fire of moderate severity increased the toxicity of PAHs in soils, whereas the high severity fire did not cause increased toxicity. On the other hand, a high severity fire resulted in a decrease of the toxicity of halogenated PAH in soils. Burn severity is an important factor regarding the risk for ecosystems and human health derived from soil contamination by PAH after wildfires.

Rey-Salgueiro et al. [128] investigated the influence of the dominant species and fire severity on PAH production in forest and shrubland fires in north-western and central Spain. The authors sampled and analysed for PAH unburnt or charred litter from four sites affected by wildfires, dominated by *Pinus pinaster*, *Pinus nigra*, *Ulex europaeus*, or *Erica arborea*. Unburnt soils and two burn severities (moderate, 200–400 °C, and high, 400–600 °C) were compared at each site. The sampling was carried out three to seven days after wildfire. The PAH analysed were fluoranthene, pyrene, benzo[*a*]anthracene, chrysene, benzo[*b*]fluoranthene, benzo[*k*]fluoranthene, benzo[*a*]pyrene, dibenzo[*ah*]anthracene, benzo[*ghi*]perylene, and indeno[1,2,3-*cd*]pyrene. The total PAH concentrations ranged from 8.5 µg kg^−^^1^ (in high severity burnt *P. pinaster* litter) to 50 µg kg^−^^1^ (in moderate severity burnt *P. pinaster* litter), well below the Dutch intervention value. These values are low compared to those reported for South Korea [105,109,119], Malaysia [112], Nigeria [116,117], Portugal [93], Russia [123,124], or California [127]. However, it must be emphasised that the referred studies include some compounds, namely two- and three-ring PAH, not determined in the present study and reported as abundant in the studies cited. The values reported here are also lower than those reported by García-Falcón et al. [111] for a burnt peri-urban woodland soil in north-west Spain, although that study includes less PAH than the present study. The values reported by Rey-Salgueiro et al. [128] are comparable to those of Vergnoux et al. [115], which included PAH not included here but sampling was performed one and three years after the wildfire. Consistent with other studies, the low molecular weight PAH (including fluoranthene, pyrene, and chrysene) were found at higher concentrations than high molecular weight PAH (including benzo[*b*]fluoranthene, benzo[*k*]fluoranthene, and benzo[*a*]pyrene) in all samples. In agreement with Chen et al. [127], for each type of litter the concentration of PAH, and especially of low molecular weight PAH, increased with moderate severity burning and decreased again with high severity burning. The lower concentrations of PAH at high severity burning compared to moderate severity burning are related to high combustion efficiency. According to the authors, the PAH concentrations are expected to decrease with time through transport or biodegradation.

Ribeiro et al. [129] investigated the presence of PAH in burnt and unburnt soils in the Caramulo range in Portugal. Two soil horizons (0–5 cm and 5–20 cm depth) were sampled from burnt and unburnt soils immediately after wildfires and then every 6 months for 2 years. The O horizon was excluded in unburnt soils while the ash layer was sampled in burnt soils in the first sampling date. The 16 EPA priority PAH were determined by GC-MS. The total PAH concentrations ranged between 0.66 and 223.2 ng g^−1^ in soils and amounted up to 13.51 ng g^−1^ in ash, in both cases clearly below the Dutch intervention value. Excepting a sample with a total concentration of 223.2 ng g^−1^, the concentrations were in line with those of Rey-Salgueiro et al. [128]. The variations of PAH concentrations with soil depth or time after wildfire did not show a consistent trend, although from six months after fire, the PAH declined over time in all soils. In contrast to most published studies, high molecular weight (4–6 ring) PAH predominated over low molecular weight (2–3 ring) PAH, the most abundant in soil samples being benzo[*k*]fluoranthene, followed by minor proportions of fluoranthene, pyrene, chrysene, benzo[*b*]fluoranthene, and indeno[1,2,3-*cd*]pyrene. Naphthalene, acenaphthylene, and acenaphthene were not detected in any sample. The authors attributed their absence to degradation and volatilisation during burning. Benzo[*a*]pyrene was present in burnt soils at a maximum concentration of 1.30 ng g^−1^, but not in unburnt soils. Only the concentration of benzo[*k*]fluoranthene exceeded the limit established by Portuguese guidelines for agricultural soils in one sample.

The literature reviewed showed an enrichment in PAH, PCB, and dioxins of burnt soils relative to unburnt soils. Usually, low molecular weight PAH prevailed over high molecular weight PAH. Transportation of PAH by the wind can lead to contamination of distant soils or water bodies. PAH, PCB, and dioxins were sporadically present in concentrations exceeding the Dutch or Canadian guidelines and had low persistence in soil, disappearing by transport or biodegradation. These compounds can be exported by runoff and reach water bodies. Some studies reported lower concentrations of pyrogenic organic pollutants in high-severity fires compared to moderate-severity fires, which was related to higher combustion efficiency.

## 5. Detoxifying Effect of Charcoal

In contrast to all that has been reviewed so far, charcoal, highly aromatic pyrogenic organic matter produced by fires, having a long residence time in soil and a high sorbing capacity, can produce a detoxifying effect. According to various publications, charcoal creates bioactive zones in the soil, where the soil surrounding charcoal is designated as “charosphere” [130,131].

In this respect, Zackrisson et al. [132] reported the ability of charcoal in once burnt boreal forest soils to adsorb phytotoxic phenolic compounds produced by the shrub *Empetrum hermaphroditum*. The effectiveness for this detoxifying function was high in charcoal younger than 100 years. Reheating deactivated older charcoal at temperatures above 450 °C can reactivate it. Furthermore, charcoal can enhance microbial activity. Keech et al. [133] compared the capacity to adsorb allelochemicals of charcoal from nine different woody plant species, using seed germination to measure this capacity. These authors found that the adsorption capacity of charcoal was significantly higher for deciduous trees than for conifers and ericaceous species. Moreover, they reported that the charcoal macroporosity was a determining factor of the adsorption capacity. Therefore, seed germination and success of establishing new tree seedlings after fire seems to be dependent on charcoal properties. These results highlight the need for conservation of broad-leaved deciduous trees in boreal forests.

DeLuca et al. [134] studied N mineralisation, nitrification, and sorption of free phenolic compounds along a fire-induced chronosequence (forest stands varying in age since the last fire) in northern Sweden. Nitrification and net N mineralisation were highest in recently burnt soils. The decline of nitrification with time since last fire could be due either to increasing cover of ericaceous shrubs (which release polyphenols that could inhibit nitrification) or to decrease in the charcoal sorption capacity. However, no significant relationships were found between nitrification rate and ericaceous vegetation cover or between nitrification rate and total polyphenol concentration. The authors argue that the decline in net N mineralisation with time may result from increasing N immobilisation or from the presence of a recalcitrant N pool rather than from inhibition. Amending the soil with glycine (an organic N source) resulted in increased ammonification but not nitrification. The addition of charcoal and glycine to soil stimulated nitrification. Moreover, the addition of charcoal significantly reduced the concentration of polyphenols. According to the authors, it is possible that the charcoal adsorbed specific phenolic compounds which otherwise would have been inhibitory to autotrophic nitrifying bacteria.

Berglund et al. [135] conducted laboratory and field studies to investigate the effect of soil amendment with activated carbon (used as a surrogate for soil charcoal) on nitrification in boreal forest soils. Glycine was added to the soils as an organic nitrogen source, both in laboratory and field experiments. Laboratory incubations showed increased net nitrification in soils amended with activated carbon, but no significant differences were observed in the field study. Significant adsorption of free phenols by activated carbon was shown in the field study. The authors suggest that the positive effect of activated carbon on nitrification in laboratory incubation could result from the adsorption of phenolic compounds, which have a generally negative effect on nitrifying bacteria, while in the field study there might have been an increase in gross nitrification but not in net nitrification.

Gundale and DeLuca [136] investigated several properties of charcoal produced in the laboratory from ponderosa pine (*Pinus ponderosa*) and Douglas-fir (*Psuedotsuga menziesii*) bark and wood at two temperatures (350 and 800 °C, which represent lower and upper thresholds for charcoal formation). They reported that all charcoal types were effective at sorbing catechin, an allelochemical identified as a root exudate of many plant species. The determined sorption capacity was in all cases more than 5 mg catechin g^−1^ charcoal. The catechin sorption capacity was significantly higher in charcoals produced at higher temperature. This fact highlights the importance for charcoal properties of charring temperature during fire events, which is related to fire intensity. Ponderosa pine wood and bark charcoals also had a significantly higher capacity to sorb catechin than Douglas-fir wood and bark charcoals at both temperatures, but these differences were minor compared to those produced by temperature. A high capacity of natural charcoals to sorb allelochemicals may contribute to a high biodiversity observed in some fire-maintained ecosystems.

MacKenzie and DeLuca [137] reported that field-collected charcoal was extremely effective at sorbing phenols from leachates of litter of *Arctostaphylos uva-ursi* (L.) Spreng., an ericaceous shrub, removing more than 80% of phenolic compounds in solution. In the same study, they found that glycine (an organic nitrogen source) stimulated significant nitrification when applied to incubated litter samples of *Carex geyeri* Boott (a sedge) but not of *Arctostaphylos uva-ursi*. However, in agreement with DeLuca et al. [134], the addition of glycine and charcoal to incubated litter of *Arctostaphylos uva-ursi* triggered nitrification, so increasing available nitrogen, which has been observed in forest soils for years after a fire. Various authors reported the increase of nitrification by charcoal [136,138,139,140]. MacKenzie and DeLuca [137] interpret their results as an evidence that increased nitrification results from adsorption by charcoal of phenols that inhibit microbes responsible for nitrification.

DeLuca and Sala [141] studied in laboratory and field experiments the nitrogen dynamics in ponderosa pine/Douglas fir forests, comparing stands not burnt for at least 69 years (designed as “unburnt”) with others that underwent at least two fires over the last 100 years (designed as “frequently burnt”). Total N in the forest floor decreased in frequently burnt soils (from 8.8 g/kg in unburnt stands to 6.2 g/kg in frequently burnt stands). In the mineral soil, there was no significant change in total N but the potentially mineralisable N (measured by anaerobic incubation) decreased in frequently burnt soils. In contrast, the net nitrification (measured both in the laboratory and in situ) and net N mineralisation (in laboratory measurements) increased significantly in frequently burnt stands relative to unburnt stands. Similar to fire-exposed soils, soils amended with charcoal showed a significant increase in the nitrification potential (measured by a short-term aerobic slurry method). The results indicate the role of charcoal in maintaining nitrification in frequently burnt stands for a long time after fire. The effect of charcoal could be related to adsorption of compounds that might inhibit nitrification.

Makoto et al. [142] investigated the dynamics of nitrogen and other nutrients after an experimental burning of low intensity in a Japanese forest of Japanese white birch (*Betula platyphylla* var. *japonica*) with understorey of dwarf bamboo (*Sasa senanensis*). These authors compared unburnt control plots, burnt plots, and burnt plots from which the charcoal was removed. In contrast to other studies, the results of this research did not indicate a stimulation of nitrification by charcoal, the nitrate extractable from the soil humus layer being highest in soils from which the charcoal was removed, from immediately after the fire up to two months later. It is worth mentioning that the charcoal in this study, produced at low temperature, had, according to the authors, a low adsorption capacity. It should also be noted that phenolics were not abundant in the forest floor, derived mainly from dwarf bamboo. In a recent review, Makoto and Koike [143] highlight the prevalence of articles that report charcoal favouring nutrient mineralisation rather than immobilisation, enhancing litter and humus decomposition in boreal forests.

Pingree et al. [144] evaluated the adsorption of phenol by field-collected and laboratory-produced charcoal. The adsorption capacity of wildfire-produced charcoal was on average 29.70 μg phenol mg charcoal^−1^ and was not affected by time since fire. The adsorption capacity of laboratory-produced charcoal increased with increasing formation temperature, ranging between 61.2 μg phenol mg charcoal^−1^ (300 °C) and 174.8 μg phenol mg charcoal^−1^ (800 °C).

In a laboratory study, Carter et al. [145] collected unburnt soil samples from Jeffrey pine (*Pinus jeffreyi* Balf.) or lodgepole pine (*Pinus contorta* Douglas) dominated areas, added charcoal derived from Jeffrey pine or lodgepole pine, and incubated the amended and unamended samples for eight weeks. The incubation conditions may be representative for low-severity burns. The authors reported that the charcoal adsorbed polyphenols from both soils. However, in contrast to other studies, the effects of charcoal amendments on potential nitrification were small; the data did not show consistent increases in nitrification potential at higher amendment levels, nor was nitrification lower in the soil amended with the less polyphenol adsorbing charcoal. The study showed that, after three weeks, the microbial respiration was generally higher in amended than in unamended soils. Nevertheless, no relationship was observed between respiration and polyphenol sorption. The adsorption of polyphenols did not seem relevant for the microbial activity and the exact mechanisms responsible for the charcoal effects were not identified.

In a similar way, Baker et al. [146] analysed charcoal produced by a historical wildfire occurred in Idaho, USA, in 1910. The study site is a wetland heavily contaminated by mine waste, where the charcoal appears as a sedimentary layer at 33–38 cm depth. The charcoal samples were analysed for As, Cd, P, Pb, Zn, Fe, and Mn and compared to the surrounding soil. The charcoal samples were enriched in all reported elements by 5 to 40 times compared to the surrounding soil. The most enriched elements were Pb, As, and Cd. The concentrations of potentially toxic elements in soil at 40–50 cm depth are at or near background levels, whereas the charcoal from these depths contains elevated concentrations of these elements. These results indicate that charcoal has an important role in controlling contaminant fate in the environment.

## 6. Summary and Research Needs

Besides affecting the physical, chemical, and biological soil properties and influencing the global carbon cycle and climate change, wildfires can produce or release substances potentially toxic to soil and ecosystems.

Numerous studies ascertain inhibitory effects of ash on seed germination and seedling growth as well as its toxicity to soil and aquatic organisms. Frequently, the substances responsible for this toxicity have not been identified.

The mobilisation of heavy metals and trace elements by fire was addressed by copious publications. Besides total concentrations, speciation, availability, and risk of exportation to water bodies have been analysed. Mercury is one of the most extensively studied elements. Upon combustion of litter or plant biomass, most of the mercury stored in them is released into the atmosphere.

Shortly after fire, some studies found PAH levels comparable to those of contaminated urban soils. In a few cases, reported concentrations of some PAH were above the environmental soil quality guidelines. Despite their resistance to degradation, various authors reported decreasing PAH concentrations in soil with time elapsed since fire. The decrease can occur through ash erosion, leaching, and volatilisation, but also by biological and photochemical degradation. Leaching and erosion losses of PAH pose a risk of contamination of surface and ground waters, which implies the possible entrance of PAH into the aquatic food chain and an impact on drinking water. In most studies, low molecular weight (2–4 rings) PAH prevailed over high molecular weight (5–6 rings) PAH in ash and burnt soils.

Finally, the possible detoxifying role of charcoal in soils affected by fire is addressed in this review. The detoxifying effect occurs mainly through adsorption of potentially toxic elements or toxic organic compounds.

This review has identified some research gaps which deserve to be filled. The need to study the vertical and lateral variation of the toxic substances generated by the fires is perceived. In the studies reviewed, the depth of soil sampling is highly variable. Some works study only the organic horizon (litter or ash); others sample only mineral soil or both organic and mineral horizons; in some cases, the ash is sampled together with the topsoil. Rarely various soil depths are analysed and the migration of pollutants in depth is studied. Even more rarely, the studies address the lateral downslope migration of these substances or their transport by the wind. Although it is common to sample different soils within an area or transect, the directions of the slope or wind are seldom taken into account.

Numerous studies report ecotoxicological effects of ash on soil organisms, but usually the substance causing the toxic effect is not identified. On the other hand, abundant research analyses heavy metals or organic pollutants in burnt soils but does not include an ecotoxicological study. It seems necessary to carry out studies that combine ecotoxicological research in burnt soils with the identification and quantification of the substances causing the toxicity.

In the case of PAH, the number of individual PAH analysed is variable. Most of the studies analyse the EPA’s 16 priority PAH; some analyse a smaller number; in one case, 29 PAH were analysed. However, some recent studies [147,148,149,150,151] question the adequacy of the 16 priority PAH to diagnose an environmental or health risk. Therefore, it is necessary to adapt the PAH analysed to the new emerging criteria for the selection of individual PAH indicative of environmental or health hazard. The analysis of other organic pollutants, such as PCB and dioxins which have rarely been studied, is also seen as necessary.

## Figures and Tables

**Table 1:** Effects of ash or burning on plant species.

Species	Effect	Cause	References
*Pinus banksiana* Lamb.	Impaired seedling survival	High pH	[15,16]
*Pinus halepensis*, *Cistus salviifolius*, *C. creticus*, *Triticum aestivum* and *Raphanus sativus*	Reduced germination and growth	High osmotic potential of the soil solution, toxic effect of certain ions, or high pH	[17,18,19]
*Pinus halepensis*, *Cistus salviifolius*, *C. creticus*, *Raphanus sativus* and *Avena sativa*	Reduced germination	High pH	[20]
*Rhus coriaria*	Germination increased by ash cover of 1 or 2 cm but inhibited by ash cover of 5 cm	Balance between the stimulating effects of heat and ethylene released by wet ash, and the inhibitory effects of the high pH and high osmotic pressure	[21]
All taxa in the soil seed bank in *Pinus halepensis* forests in Israel	Inhibition of germination	High pH	[22]
*Calluna vulgaris* and *Erica umbellata*	Lower germination rates	High pH	[23]
*Pinus pinaster*, *Pinus radiata*, and *Eucalyptus globulus*	Reduced or inhibited germination	High osmotic pressure, high pH, or toxic effects of certain ions	[24]
*Pinus sylvestris*, *P. nigra*, *P. radiata*, and *P. pinaster*	Reduced germination, no effect on seedling growth	Likely high pH	[25]
*Quercus robur*, *Q. pyrenaica*, and *Q. ilex*	No effect on germination rates		[26]
*Pinus nigra* and *P. sylvestris*	No effect of ash solutions on germination rates		[27]
*Acacia dealbata*, *A. longifolia*, *A. mearnsii*, *A. melanoxylon*, *Pinus nigra*, *P. pinaster*, *P. radiata*, *P. sylvestris*, *Quercus ilex*, *Q. pyrenaica*, *Q. robur*, and *Q. rubra*	Germination inhibited in six species (including the four species of pine), not affected in five species (including *Quercus ilex*, *Q. pyrenaica*, and *Q. robur*) and stimulated in *Q. rubra* by ash of *Ulex europaeus*	Decreased germination likely related to the sensitivity of seeds to the high osmotic pressure induced by the ash Stimulating effect of ash of *Ulex europaeus* likely related to its richness in N	[28]
*Lepidium sativum*	Reduced germination	Undetermined non-hydrosoluble phytotoxic organic substances	[29]
*Lepidium sativum* L. and *Sinapis alba* L.	Reduced root growth	Undetermined	[30]

**Table 2:** Effects of ash or burning on soil microorganisms.

Species	Effect	Cause	Reference
Soil microfungi	Growth inhibited by aqueous extracts of burnt litter	Presence of an undetermined toxic water-soluble substance	[31]
Soil bacteria	Decreased bacterial activity by soil heating	Presence of an undetermined toxic substance	[32]
Soil microorganisms	Decreased respiration rate, modified structure of the microbial community, and toxicity of extracts of burnt humus to the indicator luminescent bacteria *Vibrio fischeri*	Inhibitory compounds of low molecular mass present in the hydrophilic base fraction of DOC	[34]
Soil microorganisms	The addition of black ash increased basal respiration but did not affect biomass-C, bacteria, and fungi numbers	Organic carbon content of ash	[35]
Soil microorganisms	Microbial and fungal biomass reduced by fire treatment but not affected by ash application. Respiration rate decreased by fire treatment and increased by ash application	Decreased microbial biomass and soil respiration in the fire treatment due to fire itself, not to ash. Faster mineralisation of organic material in ash-fertilised plots	[36]

**Table 3:** Effects of ash on other soil and aquatic organisms.

Species	Effect	Cause	Reference
*Enchytraeus* sp. and *Biomphalaria glabrata*	Adverse effects on reproduction of *Enchytraeus* sp. and *Biomphalaria glabrata*	High pH and possibly unidentified compounds present in ash	[37]
*Ceriodaphnia dubia*, *Danio rerio*, and *Biomphalaria glabrata*	Acute toxicity to *C. dubia* at low concentrations, minor toxic effects to *D. rerio*, and no acute toxicity to *B. glabrata*	Presence of unidentified toxic substances	[38]
*Daphnia magna*	Three of the six types of ash tested (Australia, USA, and Canada) showed notable toxicity to *D. magna*	High pH and electrical conductivity. Mobilisation of toxic ash components at high pH	[39]

**Table 4:** Dutch and Canadian guidelines and values published by Kabata-Pendias et al. [61] for threshold concentrations of trace elements in agricultural soils (mg/kg). Data from [61,62,63].

Element	DIV ^a^	DIL ^b^	CGA ^c^	CGR ^d^	CGIA ^e^	CGIR ^f^	MAC ^g^	TAV ^h^
Ag	-	15	-	-	20	20	-	2–40
As	76	-	12	12			15–20	10–65
Ba	-	-	750	500			-	400–600
Be	-	30	4	4			10	10–300
Cd	13	-	1.4	10			1–5	2–20
Co	190	-	-	-	40	50	20–50	30–100
Cr(total)	-	-	64	64			50–200	50–450
Cr(III)	180	-	-	-			-	-
Cr(VI)	78	-	0.4	0.4			-	3–25
Cu	190	-	63	63			60–150	60–500
Hg (total)	-	-	-	-			0.5–5	1.5–10
Hg (inorganic)	36	-	6.6	6.6			-	-
Hg (organic)	4	-	-	-			-	-
Mo	190	-	-	-	5	10	4–10	5–20
Ni	100	-	45	45			20–60	75–150
Pb	530	-	70	140			20–300	50–300
Sb	22	-	-	-	20	20	10	10–20
Se	-	100	1	1			-	3–10
Sn	-	900	-	-			-	35–50
V	-	250	130	130			150	100–340
Zn	720	-	250	250			100–300	200–1500

^a^ Dutch intervention value (mg/kg) [62], ^b^ Dutch indicative levels for severe contamination (mg/kg) [62], ^c^ Canadian Environmental Quality Guidelines. Agricultural (mg/kg) [63], ^d^ Canadian Environmental Quality Guidelines. Residential/parkland (mg/kg) [63], ^e^ Canadian Environmental Quality Guidelines. Interim remediation criteria for soil (1991). Agricultural (mg/kg) [63], ^f^ Canadian Environmental Quality Guidelines. Interim remediation criteria for soil (1991). Residential/parkland (mg/kg) [63], ^g^ Ranges of maximum allowable concentrations in agricultural soils (mg/kg) [61], ^h^ Trigger action value for trace metals in agricultural soils (mg/kg) [61].

**Table 5:** Total mercury concentrations in burnt and unburnt soils, litter and ash (µg kg^−1^).

	Unburnt	Burnt	Reference
Litter or ash	81–96	24–49	[69]
Mineral soil	3–47	3–49	
Litter or ash	58–208	14–73	[70]
Mineral soil	30–68	12–63	
Litter or ash	38–75	5–38	[71]
Mineral soil	9–17	9–18	
Mineral soil	4–176	2–349	[72]
Litter or ash *	30–290		[73]
Mineral soil *	10–350	1–80	
Litter or ash	84	5–26	[74]
Mineral soil	92	80	
Litter or ash		100–150	[75]
Mineral soil	86–88	61–67	
Litter or ash		20–400	[66]
Mineral soil	3100	2200–4900	

* In this case, burnt means recently burnt soils and unburnt means old burnt soils.

**Table 6:** Concentrations of various trace elements in some burnt and unburnt soils, litter, and ash (mg kg^−1^).

		Mn	Fe	Cu	Pb	Cd	Zn	Ni	Cr	As	Co	V	References
Unburnt Burnt	Total	1100	22,000	8	28	0.40							[76]
	Available	30	30	0.55	0.4	0.12							
	Total	1200–1300	23,000–24,000	9–10	30	0.40–0.45							
	Available	50	40–45	0.65–0.70	0.5–0.6	0.16–0.17							
Burnt	Litter			7.7–501	20.2–246		8.5–136	3.1–37.0	3.8–18.4				[54]
	Organic soil			2.5–203	1.4–333		3.5–865	0.4–76.7	2.0–67.1				
Unburnt Soil				16–27	18–42.5	0.4–6.5	14.5–68.7						[77]
Burnt Soil				16–34	95–162	0.0–5.6	168–219						
Ash					1.0–36.5								[78]
Burnt soil					4.3–23.0								
Ash		107–190		3.1–4.3			31.9–68.2						[79]
Burnt soil, 5 months		172–449		0.9–2.8			5.9–17.6						
Burnt soil, 11 months		15.9–38.5		1.7–4.7			3.4–5.5						
Unburnt soil, 11 months		2.8–242		1.1–4.6			3.2–11						
Ash		328–4250	5500–68,000	24–2010	6–617	0.03–16	27–2820	0.3–107	8–1130	1–161			[80]
Burnt soil		429–2050	17,400–134,000	20–55	14–97	0.06–0.36	42–149	7.3–59	13–164	1–18			
Unburnt soil		460–1100	15,700–47,900	12–56	20–33	0.03–0.11	48–67	3.5–83	8.6–154	1–13			
Ash		67–598		22–54	52–122	0.12–0.49		12–32			1.8–4.8	25–62	[55]
Burnt soil		13–177		15–32	17–132	0.06–0.18		8–18			1.1–4.1	29–57	
Unburnt soil		12		19	40	0.05		5			1.8	20	
Ash		139–317	1191–2996		3.4–6.6	0.1–0.3	29.9–85.5	1.3–5.7	12.8–19.9			9.6–21	[38]
Burnt soil		46–110	33–61		15–17	4.0–7.3	17.9–25.8	<<<	164–270			107–160	
Ash		330–2790		56–207	1.8–14.9	0.02–0.22	221–555	9.5–37.6	3.3–9.0	21–260	5.2–12.6		[81,82]
Burnt soil		70–7000		6–90	8–52	0.01–0.7	12–611	5–26	15–61	12–544	3–16		
Unburnt soil		85–560		14–59	12–76	0.03–0.37	22–328	7–23	23–57	19–185	4–25		

<<<: below detection limit.

**Table 7:** Maximum concentrations of As, Pb, Sb, Cu, Zn, and Cr in ash from burnt areas of southern California after wildfires in 2007 and 2009 and USEPA preliminary remediation goals. Data from [83,84].

Element	Concentration in Ash, mg/kg	USEPA PRG (Soil), mg/kg *
As	≤140	0.4–0.62
Pb	≤344	150–400
Sb	≤32	31
Cu	≤1370	3100
Zn	≤2800	23,000
Cr	≤354	210 (30 mg/kg of Cr(VI))

* PRG = Preliminary Remediation Goal.

**Table 8:** Total concentrations of trace elements in litter (unburnt), ash, and soil (3–8 cm depth) samples at three fire severities 10 weeks after a wildfire south-west of Sydney, Australia. Data from [86].

Element	Litter	Ash			Soil			
		Low Severity	High Severity	Extreme Severity	Unburnt	Low Severity	High Severity	Extreme Severity
B, mg kg^−^^1^	6.0	2.9	3.4	4.1	0.8	0.6	1.0	1.3
Cu, mg kg^−1^	4.3	6.3	5.1	5.3	1.9	2.4	1.7	1.2
As, mg kg^−1^	0.1	5.1	4.0	7.3	5.1	3.9	3.8	4.2
Cd, µg kg^−1^	102.1	88.3	79.6	82.5	31.0	23.2	32.5	25.1
Hg, µg kg^−1^	72.6	1.7	1.8	4.2	5.4	5.8	6.7	6.9
Pb, mg kg^−1^	0.6	10.7	12.6	21.4	12.9	10.5	14.2	13.5

**Table 9:** Dutch and Canadian guidelines for PAH, PCB, and PCDD/F concentrations in soils.

Substance	DIV ^a^	CGA ^b^	CGR ^c^
PAH (total), mg kg^−1^	40	-	-
Naphthalene, mg kg^−1^ *	-	0.013	0.013
Anthracene, mg kg^−1^ *	-	2.5	2.5
Phenanthrene, mg kg^−1^ *	-	0.046	0.046
Fluoranthene, mg kg^−1^ *	-	50	50
Pyrene, mg kg^−1^ *	-	0.1	10
Benzo[*a*]anthracene, mg kg^−1^ *	-	0.1	1
Benzo[*b*]fluoranthene, mg kg^−1^ *	-	0.1	1
Benzo[*k*]fluoranthene, mg kg^−1^ *	-	0.1	1
Benzo[*b + j + k*]fluoranthene, mg kg^−1^ *	-	0.1	1
Dibenz[*a*,*h*]anthracene, mg kg^−1^ *	-	0.1	1
Indeno[1,2,3-*c*,*d*]pyrene, mg kg^−1^ *	-	0.1	1
Benzo[*a*]pyrene, mg kg^−1^ *	-	20	20
PCB, mg kg^−1^		0.5	1.3
PCDD/F, ng TEQ ** kg^−1^	180	4	4

^a^ Dutch intervention value [62], ^b^ Canadian Environmental Quality Guidelines. Agricultural [63], ^c^ Canadian Environmental Quality Guidelines. Residential/parkland [63], * The values presented for PAH are intended for protection of the environment, not of human health, ** Toxic equivalent quantity.

**Table 10:** Concentrations of PAH in airborne particulate matter from bush fire (mg/kg). Data from [106].

FLU	PYR	B*a*A	CHR	B*b*F	B*k*F	B*a*P	D*b*A	B*ghi*P	IP	COR
200	128	38.1	125	6.0	20.7	194	12.2	8.2	19.5	26.0

FLU: fluoranthene, PYR: pyrene, B*a*A: benz[*a*]anthracene, CHR: chrysene, B*b*F: benzo[*b*]fluoranthene, B*k*F: benzo[*k*]fluoranthene, B*a*P: benzo[*a*]pyrene, D*b*A: dibenz[*a,h*]anthracene, B*ghi*P: benzo[*ghi*]perylene, IP: indeno[*cd*]pyrene, COR: coronene.

**Table 11:** Concentrations of PAH in surface soils immediately after a wildfire (W-A) and two years after a wildfire (W-2), µg/kg. Data from [107].

Compounds	W-A	W-2
Alkylnaphthalenes	50	-
Fluorene and derivatives	96	-
Phenanthrene	10	-
Alkylphenanthrenes	1198	22
4/5 cycles PAH *	20	-

* pyrene, chrysene, benz[*a*]anthracene, benz[*c*]pyrene, perylene.

**Table 12:** Mean concentrations ± standard errors of ∑20 polycyclic aromatic hydrocarbons (PAH), and ∑14 polychlorinated biphenyls (PCBs) in the control plots (*n* = 4) and in the soil surface horizons 1 y after application of 8 Mg ha^−^^1^ wood ash. The percentage of low molecular weight PAH (LMPAH) and lower chlorinated PCB (LCPCB) is given in parentheses. Data from [104].

	∑20 PAH (µg kg^−^^1^)		∑14 PCB (µg kg^−^^1^)	
Horizon	Control	+Wood Ash	Control	+Wood Ash
Oi	1250 ± 330 (49)	1390 ± 270 (46)	25.4 ± 5 (47)	21.1 ± 5 (43)
Oe	1350 ± 120 (42)	7660 ± 2960 (30)	33.1 ± 3 (31)	21.3 6 ± (40)
Oa	1390 ± 220 (38)	8050 ± 4120 (29)	32.5 ± 8 (27)	33.7 ± 5 (26)
Ah	527 ± 81 (40)	492 ± 97 (47)	5.5 ± 0.5 (29)	6.0 ± 1 (18)

**Table 13:** Concentrations of PCDD/F and PAH in the burnt and unburnt soils 1, 5, and 9 months after a forest fire. Data from [105,109].

Compounds	1 Month	5 Months	9 Months	Control
Total PCDD/F, ng kg^−1^	197–368	98–130	37–168	142
PCDD/F, ng TEQ kg^−1^	2.60–4.82	0.68–1.42	0.32–1.35	0.99
Total PAH, ng g^−1^	942–1570	153–304	205–481	49.4
Naphthalene, ng g^−1^	268–420	3.21–39.1	1.18–63.5	4.91
Acenaphthylene, ng g^−1^	12.1–126	0.40–4.27	3.94–43.1	0.17
Acenaphthene, ng g^−1^	5.88–31.5	0.78–1.88	8.96–17.7	1.31
Fluorene, ng g^−1^	77.4–105	5.09–25.9	9.10–21.8	4.77
Phenanthrene, ng g^−1^	169–299	44.9–105	40.7–175	7.14
Anthracene, ng g^−1^	138–202	12.5–39.6	4.61–29.4	1.17
Fluoranthene, ng g^−1^	54.8–234	22.9–41.6	19.0–53.9	5.11
Pyrene, ng g^−1^	27.1–116	17.6–20.3	11.6–33.8	3.55
Benz[*a*]anthracene, ng g^−1^	8.39–61.3	9.37–6.16	9.79–12.9	2.90
Chrysene, ng g^−1^	12.7–42.2	7.68–8.69	13.5–37.1	4.72
Benzo[*b*]fluoranthene, ng g^−1^	6.07–33.7	5.23–8.57	8.46–16.0	5.16
Benzo[*k*]fluoranthene, ng g^−1^	0.93–7.49	1.00–1.92	5.71–10.7	3.92
Benzo[*a*]pyrene, ng g^−1^	3.93–23.9	1.83–2.19	4.78–6.46	1.63
Indeno[1,2,3-*c*,*d*]pyrene, ng g^−1^	10.9	1.68–2.69	14.3	1.22
Dibenzo[*a,h*]anthracene, ng g^−1^	3.82	1.06–1.20	n.d.	0.66
Benzo[*ghi*]perylene, ng g^−^^1^	11.6	1.65–2.99	4.70–5.38	1.03
